# Positive feedback between the T cell kinase Zap70 and its substrate LAT acts as a clustering-dependent signaling switch

**DOI:** 10.1016/j.celrep.2021.109280

**Published:** 2021-06-22

**Authors:** Elliot Dine, Ellen H. Reed, Jared E. Toettcher

**Affiliations:** 1 Department of Molecular Biology, Princeton University, Princeton, NJ 08544, USA; 2 IRCC International Research Collaboration Center, National Institutes of Natural Sciences, 4-3-13 Toranomon, Minato-ku, Tokyo 105-0001, Japan; 3 Lead contact

## Abstract

Protein clustering is pervasive in cell signaling, yet how signaling from higher-order assemblies differs from simpler forms of molecular organization is still poorly understood. We present an optogenetic approach to switch between oligomers and heterodimers with a single point mutation. We apply this system to study signaling from the kinase Zap70 and its substrate linker for activation of T cells (LAT), proteins that normally form membrane-localized condensates during T cell activation. We find that fibroblasts expressing synthetic Zap70:LAT clusters activate downstream signaling, whereas one-to-one heterodimers do not. We provide evidence that clusters harbor a positive feedback loop among Zap70, LAT, and Src-family kinases that binds phosphorylated LAT and further activates Zap70. Finally, we extend our optogenetic approach to the native T cell signaling context, where light-induced LAT clustering is sufficient to drive a calcium response. Our study reveals a specific signaling function for protein clusters and identifies a biochemical circuit that robustly senses protein oligomerization state.

## In brief

Dine et al. study how different modes of molecular organization contribute to cell signaling using the kinase Zap70 and its substrate LAT as a model system. Optogenetic manipulation reveals that LAT:Zap70 clusters—but not dimers—trigger potent signaling via localized positive feedback among LAT, Zap70, and Src-family kinases.

## Graphical abstract

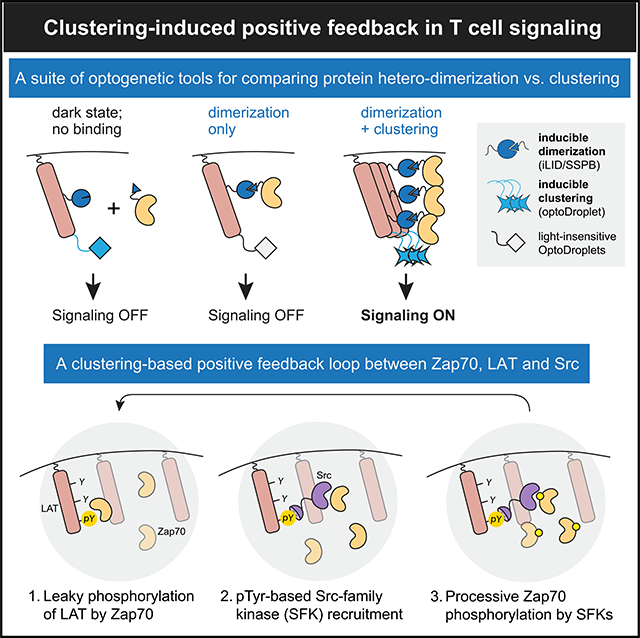

## INTRODUCTION

Many cell signaling processes involve the dynamic assembly and disassembly of protein clusters. In some cases, such as Notch/Delta complexes (Nandagopal et al., 2018) and death receptor signaling (Pan et al., 2019), clusters may emerge due to higher-order oligomerization of the receptor itself upon ligand binding. In others (e.g., receptor tyrosine kinases; the Wnt signalosome), clustering emerges from the convergence of adaptor proteins that bind via modular, multivalent interaction domains to form liquid or gel-like condensates in response to ligand stimulation (Case et al., 2019). Recent advances in imaging have established that protein clustering can accompany signaling pathway activation *in vivo* (Banjade and Rosen, 2014; Gammons and Bienz, 2018; Liang et al., 2018), and biochemical reconstitution experiments demonstrate kinase-triggered clustering of minimal sets of components *in vitro* (Houtman et al., 2006; Li et al., 2012; Su et al., 2016), suggesting that mesoscale protein assemblies are fundamental to eukaryotic cell signaling.

**Figure 1.**
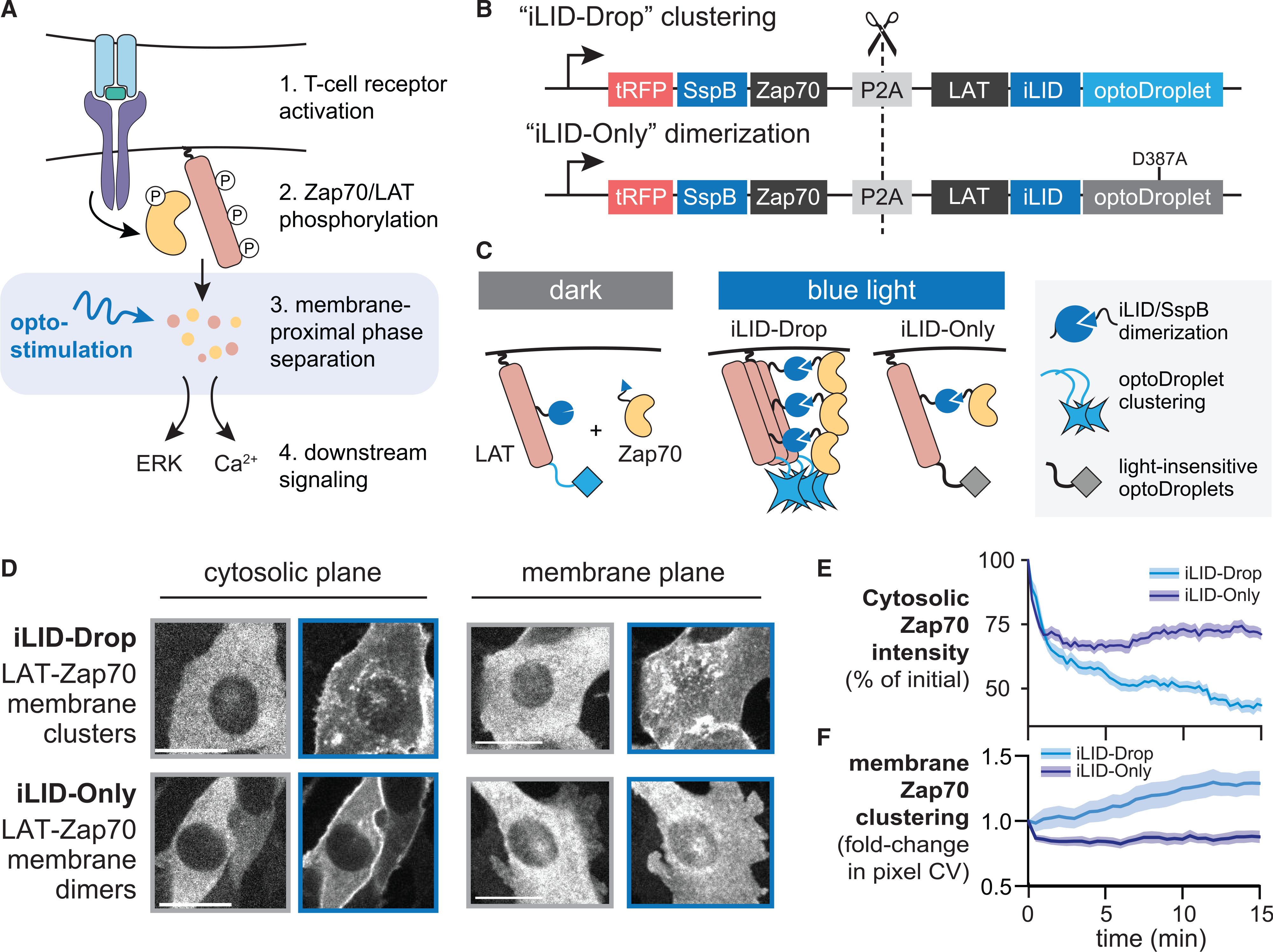
Development of optogenetic systems to compare Zap70:LAT oligomerization states (A) Schematic of TCR signaling and how optogenetic approaches can plug in at the step of Zap70:LAT clustering; see Abraham and Weiss (2004). (B) Design of the optogenetic constructs to compare dimerization and clustering of Zap70 and LAT. tRFP, TagRFP. (C) Schematic of protein complexes formed by light stimulation for the optogenetic constructs in (B). (D) tRFP-SSPB-Zap70 localization in NIH 3T3 cells. Images were taken prior to blue light illumination (gray border) or 5 min after illumination (blue border). Scale bars, 20 μm. (E) Quantification of cytosolic tRFP-SSPB-Zap70 fluorescence after illumination in both iLID-Only and iLID-Drop cells. (F) Quantification in change of the coefficient of variation (CV) of tRFP intensity for images taken in the membrane plane during 15 min of blue light illumination. For (E) and (F), envelope shows mean + SEM for at least 20 cells in each condition. See also Figure S1 and Videos S1 and S2.

T cell receptor (TCR) signaling has emerged as a key model system for understanding the signaling roles of protein clusters, as these assemblies are formed at multiple signaling steps in an activated T cell (Dustin and Groves, 2012). Within seconds after stimulation, the TCR itself forms clusters at the site of contact with an antigen-presenting cell, leading to the recruitment of downstream effectors including the kinases Lck and Zap70. Lck and Zap70 initially co-cluster with the TCR and within minutes form peripheral clusters with downstream adaptor proteins such as the linker for activation of T cells (LAT) (Lo et al., 2018; Pageon et al., 2016). Recent *in vitro* studies have demonstrated that Zap70 phosphorylation of LAT tyrosine residues is sufficient for liquid-liquid phase separation due to interactions between these phospho-tyrosines and other multivalent signaling proteins: Grb2, SOS, and PLCγ (Houtman et al., 2006; Kortum et al., 2013; Su et al., 2016) (Figure 1A). Yet it is still unknown whether clusters are important for signaling or whether they simply arise as a byproduct of multivalent interactions. Classic experiments to abolish clustering (e.g., mutating the phosphorylatable tyrosine residues on LAT) also prevent recruitment of cytosolic signaling factors (Zhang et al., 2000), making it impossible to conclude that clustering plays a specific role. The difficulty of performing such separation-of-function studies is a common challenge encountered when studying the functional role of protein condensates in cells (Alberti et al., 2019).

These considerations leave us with a major unanswered question about the role of protein phase separation in T cell signaling; do Zap70:LAT clusters play a direct role in shaping signaling responses, or would simpler interactions (e.g., an equal number of Zap70:LAT heterodimers) lead to an equivalent outcome? The recent development of chemical biology and optogenetic tools for inducing protein clustering offers a potential way to answering both these questions (Bracha et al., 2018; Bugaj et al., 2013; Dine et al., 2018; Nakamura et al., 2018; Shin et al., 2017; Spencer et al., 1993; Taslimi et al., 2014). By triggering the assembly of clusters containing selected proteins of interest and comparing to other forms of molecular interaction, we might directly test for the functional consequences of clustering. Such user-defined “signalosomes” could also prove useful to the synthetic biologist to confer specific signal processing functions to an engineered cell (Reinkemeier et al., 2019; Zhao et al., 2019).

Here, we develop tools to relate precisely the formation of specific Zap70:LAT assemblies to a cell’s resulting signaling state (Figure 1A). We engineered optogenetic variants of Zap70 and LAT whose one-to-one dimerization or assembly into clusters could be switched with a single point mutation. We expressed these variants as a minimal synthetic system in non-T cells to answer whether and how Zap70:LAT assemblies may activate downstream signaling pathways even when divorced from upstream inputs (and clusters). Remarkably, Zap70:LAT clusters were fully competent to trigger downstream Erk and calcium signaling in mouse and human fibroblasts, even in the absence of any other T-cell-specific factors, whereas Zap70:LAT heterodimers produced no signaling response. Subsequent experiments and computational modeling revealed that clustering-induced signaling requires a 3-component positive feedback loop among Zap70, its substrate LAT, and an Src-family kinase (SFK) whose recruitment to LAT enables further activation of co-clustered Zap70. This positive feedback allows for amplification of weak stimuli into full-fledged signaling responses. We also extend our optogenetic strategy to Jurkat T cells, where light-induced LAT clusters are sufficient to trigger calcium signaling. Our results suggest that the dual ability of Src to bind phospho-tyrosines and phosphorylate nearby proteins can act as a robust clustering-based signaling switch, both for endogenous signaling processes and in synthetic kinase-based circuits.

## RESULTS

### An optogenetic platform for directing Zap70:LAT dimerization versus condensation

Our first goal was to create optogenetic tools that could be used to acutely trigger distinct modes of interaction between Zap70 and LAT, forming either one-to-one Zap70:LAT heterodimers or higher-order clusters of heterodimers upon illumination. Ideally, such a system would enable the experimentalist to toggle between dimers and clusters of dimers without changing any other parameters of the system (Figures 1B and 1C) and would bypass the requirements of TCR and LCK to bridge those two proteins together (Lo et al., 2018). To accomplish this goal, we outfitted LAT with two optogenetic systems to independently control its dimerization with Zap70 versus assembly into higher-order clusters. For Zap70:LAT dimerization, we turned to the iLID-SspB system (Guntas et al., 2015), which forms one-to-one heterodimers with a binding affinity of ~100 nM in response to blue light (Guntas et al., 2015; Hope et al., 2020). For LAT clustering, we took advantage of the optoDroplet system, which can be used to trigger membrane-localized protein droplets upon blue light stimulation (Dine et al., 2018; Shin et al., 2017). Crucially, a single point mutation in the Cry2 component of optoDroplets (Cry2 D387A) renders it completely insensitive to blue light, preventing cluster formation (Bugaj et al., 2013; Liu et al., 2008). We thus engineered two DNA constructs: one that expresses a LAT-iLID-optoDroplet fusion protein with a fluorescent, SspB-tagged Zap70 (termed “iLID-Drop”) and one that is identical except for the light-insensitive point mutation in the optoDroplet system (termed “iLID-Only”) (Figures 1B and 1C). We reasoned that this matched pair of systems constituted an ideal test case because Zap70:LAT dimerization would be controlled by identical iLID-SspB interactions in both cases, with only the additional clustering of Zap70:LAT heterodimers depending on the functionality of the optoDroplet system.

We initially set out to test whether the iLID-Drop and iLID-Only tags could indeed drive different forms of Zap70:LAT interactions. We transduced NIH 3T3 cells with lentiviral vectors expressing one or the other, sorted them for the same TagRFP levels to ensure matched expression in both cell lines (Figures S1A–S1C), and imaged the resulting cell lines by confocal microscopy. We observed rapid cytosolic depletion of TagRFP fluorescence upon illumination in both iLID-Only and iLID-Drop cells, consistent with recruitment of cytosolic TagRFP-SspB-Zap70 to membrane-localized LAT-iLID (Figures 1D and 1E: Video S1). Only iLID-Drop cells exhibited nucleation and growth of small-membrane-localized TagRFP clusters (Figure 1D; Videos S1 and S2), an effect that could be quantified by measuring the variance of TagRFP pixel intensities at the plasma membrane over time (Figure 1F). The small size and rapid motion of cellular iLID-Drop clusters made it challenging to quantify their size distribution and material state. Thus, although optoDroplet-based constructs have produced liquid-like protein droplets in prior studies (Dine et al., 2018; Shin et al., 2017), we refer to LAT-Zap70 “clusters” throughout this study due to uncertainty in their material properties.

Despite similar initial kinetics of cytosolic depletion between cell lines, we observed some additional cytosolic depletion of Zap70 in iLID-Drop cells on the same timescale as membrane clustering (Figure 1E), suggesting that Zap70:LAT clusters may also increase Zap70’s retention at the membrane. Nevertheless, any differences in Zap70 cytosolic depletion were minor compared to the variability in expression levels between cells (compare cells in Figure 1D; lower-left panels). Overall, our results indicate that both the iLID-Drop and iLID-Only systems recruit Zap70 to LAT, but only iLID-Drop induces the formation of membrane-localized Zap70:LAT clusters. These differences in molecular organization are achieved using a single point mutation and at identical expression levels, thereby providing a controlled platform for assessing the functional consequences of clustering.

### Zap70:LAT clusters, but not heterodimers, activate downstream signaling pathways

**Figure 2.**
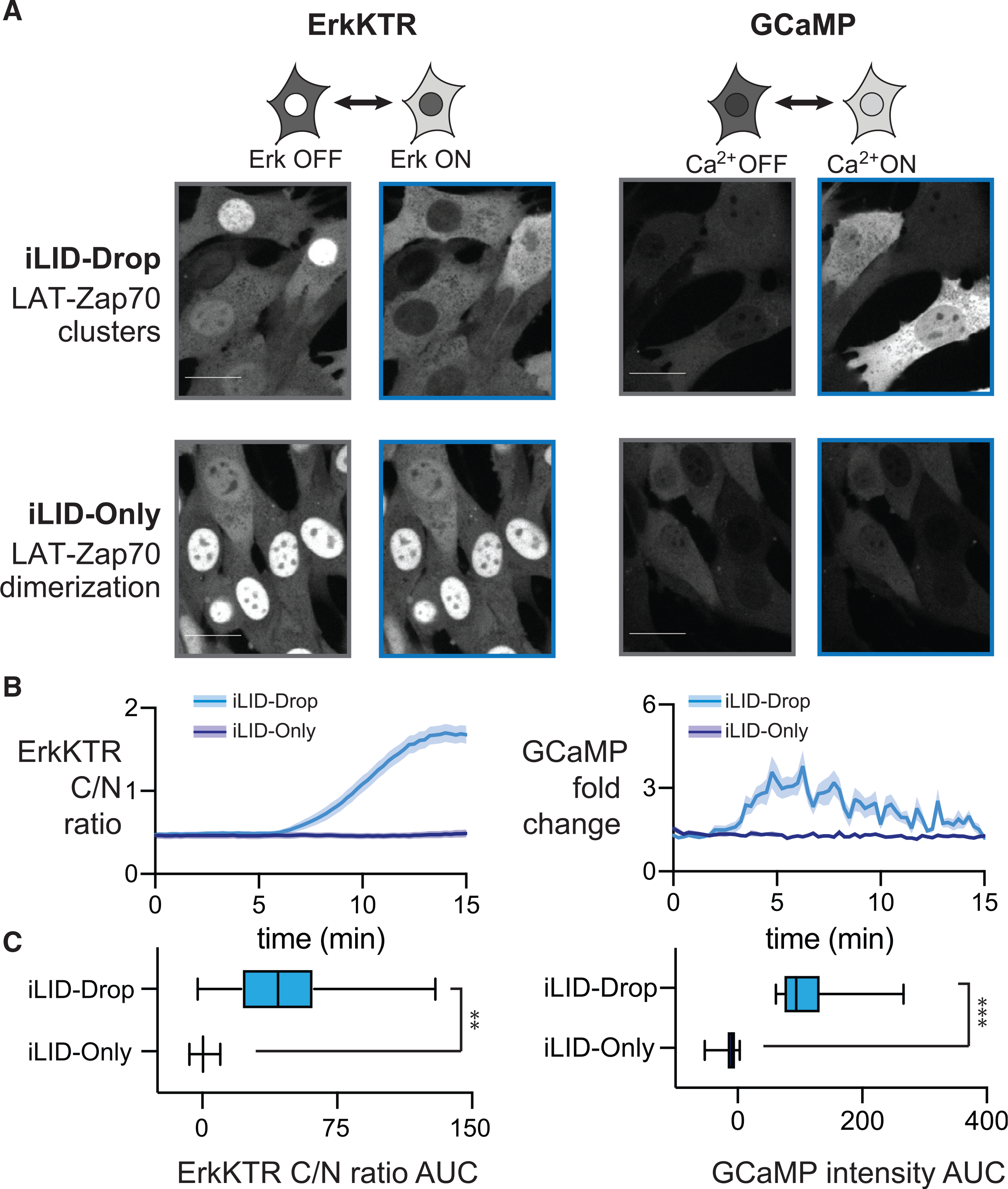
Clustering but not heterodimerization of Zap70 and LAT induces signaling (A) Images of ErkKTR-irFP and GCaMP in iLID-Only and iLID-Drop cells under illuminated (blue) and dark (gray) conditions. Scale bar, 20 μm. (B and C) Quantification of ErkKTR localization and GCaMP fluorescence for iLID-Only and iLID-Drop cells. Data show mean + SEM time courses (B) and AUC (C) for n ≥ 50 cells. For (C), boxes represent 25th–75th percentile, and whiskers show the minimum and maximum values. Statistical significance computed from four independent experiments using Student’s t test; **p < 0.01 and ***p < 0.001. See also Videos S3 and S4.

How does dimerization versus clustering of LAT and Zap70 affect the activation of downstream signaling pathways? To address this question, we set out to monitor downstream signaling in iLID-Only and iLID-Drop fibroblasts. Fibroblasts are an ideal cellular context for this study, as they lack T-cell-specific components, including the TCR and Lck, that can trigger clustering and activation of Zap70 independently of our optogenetic systems (James and Vale, 2012). However, fibroblasts still harbor intact downstream mitogen-activated protein kinase (MAPK) and calcium signaling for monitoring downstream cellular responses. We expressed the iLID-Drop and iLID-Only systems in NIH 3T3 mouse fibroblasts that were also engineered to express live-cell biosensors of downstream signaling: the Erk kinase translocation reporter (ErkKTR) and GCaMP6f (Figures 2A and 2B). The ErkKTR leaves the nucleus upon activation of Erk signaling (Regot et al., 2014), while GCaMP6f (GCaMP) becomes much brighter upon release of Ca^2+^ from stored vesicles (Dong et al., 2017).

We first generated a single NIH 3T3 cell line expressing both infrared fluorescent protein (iRFP)-tagged ErkKTR and GCaMP and then transduced and sorted for identical expression levels of either our red fluorescent Zap70:LAT iLID-Drop or iLID-Only constructs (Figure S1C). Both cell lines were plated, washed and starved in serum-free media for 2 h, and monitored for Erk and calcium responses after blue light stimulation (Figure 2A). Light-stimulated iLID-Drop cells exhibited near-complete export of ErkKTR-irFP from the nucleus and repeated spikes of GCaMP fluorescence, indicative of strong Erk and calcium signaling responses (Figures 2A and 2B; Video S3). No such responses were observed in iLID-Only cells, despite similar light-induced translocation of Zap70 to the cell membrane (Figures 2A and 2B; Video S4). We used the area under the curve (AUC) of biosensor activity in each cell to quantify and compare responses, revealing significant increases for both Erk and calcium signaling in iLID-Drop cells as compared to iLID-Only cells (Figure 2C). Calcium response dynamics matched those previously reported in T cells, reaching maximum levels in 2–3 min (Dong et al., 2017; Houtman et al., 2005). In contrast, Erk pathway activity was maximal within 10 min, matching prior optogenetic experiments done in fibroblasts (Dine et al., 2018; Toettcher et al., 2013) but slower than the 2-min timescale reported for T cells (Houtman et al., 2005), possibly reflecting additional sources of feedback or gain unique to T cells (Das et al., 2009; Huang et al., 2019). Membrane-localized clusters of Zap70 and LAT are thus sufficient to trigger Erk and calcium signaling responses even in non-T cells, whereas Zap70:LAT heterodimers are not.

### Phosphorylation and activation of Zap70 is the key clustering-dependent step

**Figure 3.**
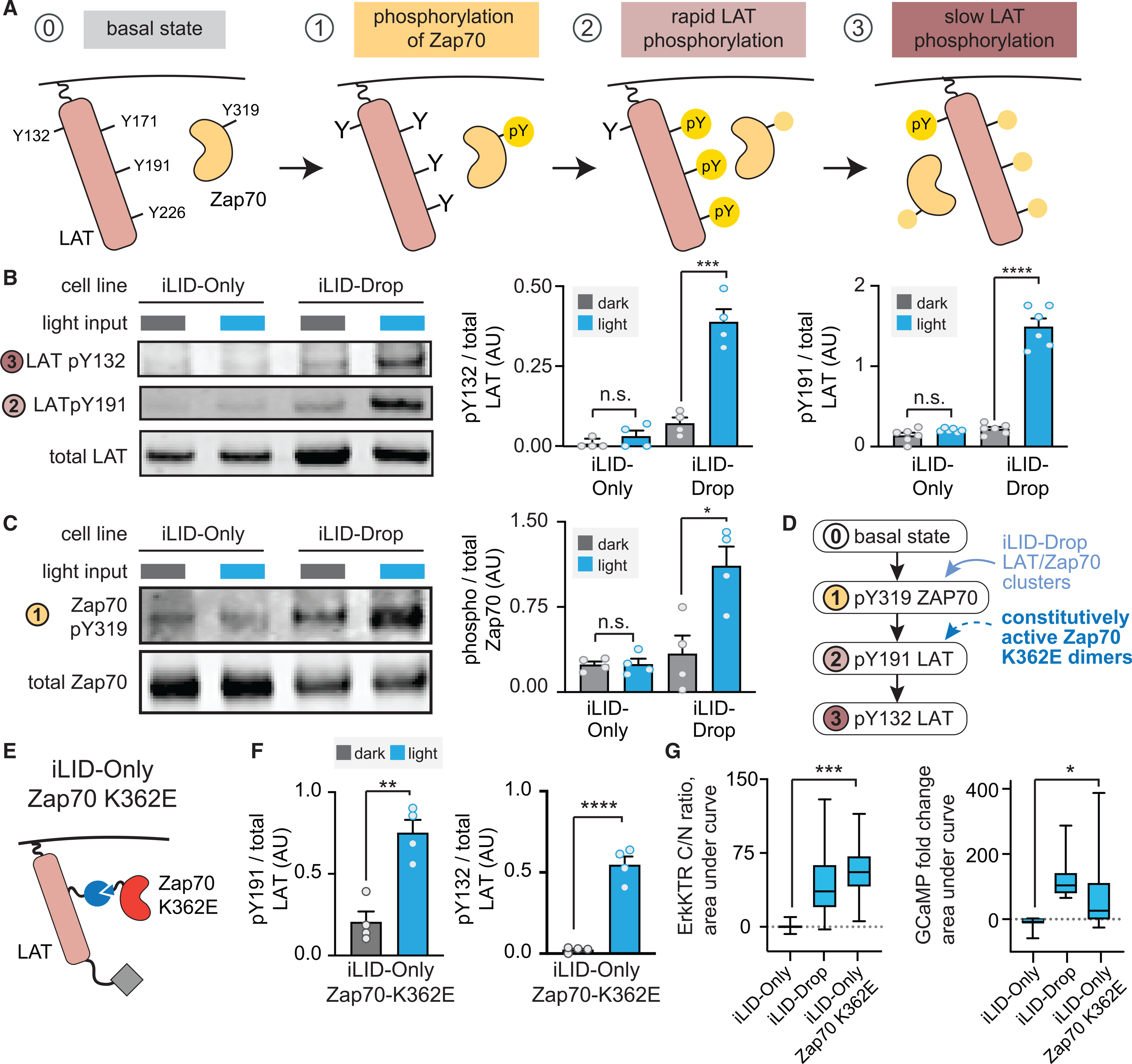
Clustering is required for light-induced Zap70 and LAT phosphorylation (A) Schematic of the Zap70:LAT interaction. Zap70 is phosphorylated on Tyr319 and then phosphorylates LAT rapidly at Tyr171, 191, and 226 and slowly at Tyr132. (B and C) Western blots and quantification of phospho-LAT Y132 and Y191 (B) and pY319-Zap70 (C) in the dark and after 20 min of blue light stimulation. (D) Schematic showing predicted responses to optogenetic clustering of LAT and Zap70 versus dimerization between LAT and Zap70^K362E^, where the Zap70 mutation could potentially enable LAT phosphorylation from dimers alone. (E) Cartoon of iLID-Only Zap70 K362E. (F) Quantification of LAT pY191 and pY132 in cells expressing iLID-Only Zap70 K362E. (G) Quantification of the integrated signaling response from ErkKTR-irFP (C/N ratio) and GCaMP (fold-change). Boxes represent 25th–75th percentile, center line shows the mean, and whiskers show minimum and maximum values. n ≥ 30 data points are shown from three different experiments. Statistical comparisons were performed using Student’s t test across all independent biological replicates. *p < 0.05, **p < 0.01, ***p < 0.001, and ****p < 0.0001. See also Figure S2 and Video S5.

We next sought to identify the biochemical steps that are activated by Zap70:LAT clustering to trigger downstream signaling. Membrane clusters have been suggested to play many distinct and separable functions, such as enhancing reaction rates by increasing local concentration, excluding negative regulators to locally increase the levels of phosphorylated species, or even altering the processivity of a kinase for its substrate during multi-site phosphorylation (Dine and Toettcher, 2018; Mayer and Yu, 2018; Shin and Brangwynne, 2017). As a first step toward identifying the mechanism for clustering-induced signaling, we monitored each of the steps normally associated with Zap70/LAT activation (Figure 3A). During T cell activation, the Zap70 kinase is first activated by phosphorylation at Tyr319. Activated Zap70 then phosphorylates LAT on four sites, three of which (Tyr171, Tyr191, and Tyr226) are rapidly phosphorylated and one of which (Tyr132) is phosphorylated more slowly and has been proposed to serve as the kinetic proofreading step for responding only to high-affinity TCR-ligand interactions (Bilal and Houtman, 2015; Houtman et al., 2005; Lo et al., 2019; Zhang et al., 2000).

We quantified Zap70 Tyr319, LAT Tyr191, and LAT Tyr132 phosphorylation under dark and illuminated conditions. All three sites were phosphorylated in a light-dependent manner in iLID-Drop cells but not in iLID-Only cells, suggesting that clustering is required even for the top-most phosphorylation event in the cascade: phosphorylation and activation of Zap70 itself (Figures 3B and 3C). Clustering-specific phosphorylation of Zap70, LAT, and downstream signaling proteins could also be observed in human-derived HEK293T cells, demonstrating that similar conclusions could be derived from multiple cell lines of either mouse or human origin (Figure S2). We also mutated Zap70 Tyr319 to phenylalanine and found that it abolished light-dependent responses in illuminated iLID-Drop cells (Figures S3A and S3B), consistent with prior reports that Zap70 phosphorylation is required for its activation and downstream signaling (Di Bartolo et al., 1999; Williams et al., 1999).

Our data indicate that clustering is required for the initial step of Zap70 phosphorylation and activation, but is this its only role? We reasoned that if clustering is required only for Zap70 activation, then a constitutively active Zap70 variant should be able to elicit a full signaling response upon dimerization in iLID-Only cells (Figure 3E). We thus established an iLID-Only NIH 3T3 cell line using a previously characterized Zap70 allele, Zap70^K362E^, that exhibits weak constitutive activity even in the absence of its phosphorylation (Figure 3E) (Lo et al., 2018). Light stimulation of iLID-Only Zap70^K362E^ cells triggered LAT phosphorylation at all tyrosine residues tested (Figure 3F), and we observed downstream signaling that generally matched what was observed in iLID-Drop cells (Figure 3G; Video S5). It is also possible that some subtle signaling differences remain between iLID-Only Zap70^K362E^ and iLID-Drop cells, such as a reduced calcium signaling response (Figure 3G), potentially reflecting contributions from other clustering-dependent regulatory mechanisms (Su et al., 2016). Nevertheless, these data demonstrate that light-induced clustering is required for signal initiation in the minimal Zap70/LAT module, and this requirement can be bypassed by a constitutively active Zap70. These data also constitute an important control, ruling out the possibility that the iLID-Only system is trivially unable to trigger downstream signaling (e.g., if the complexes induced by iLID-SspB dimerization were somehow incapable of supporting Zap70-to-LAT phospho-transfer).

### Clustering-induced Zap70 activation requires both a functional kinase and a substrate

**Figure 4.**
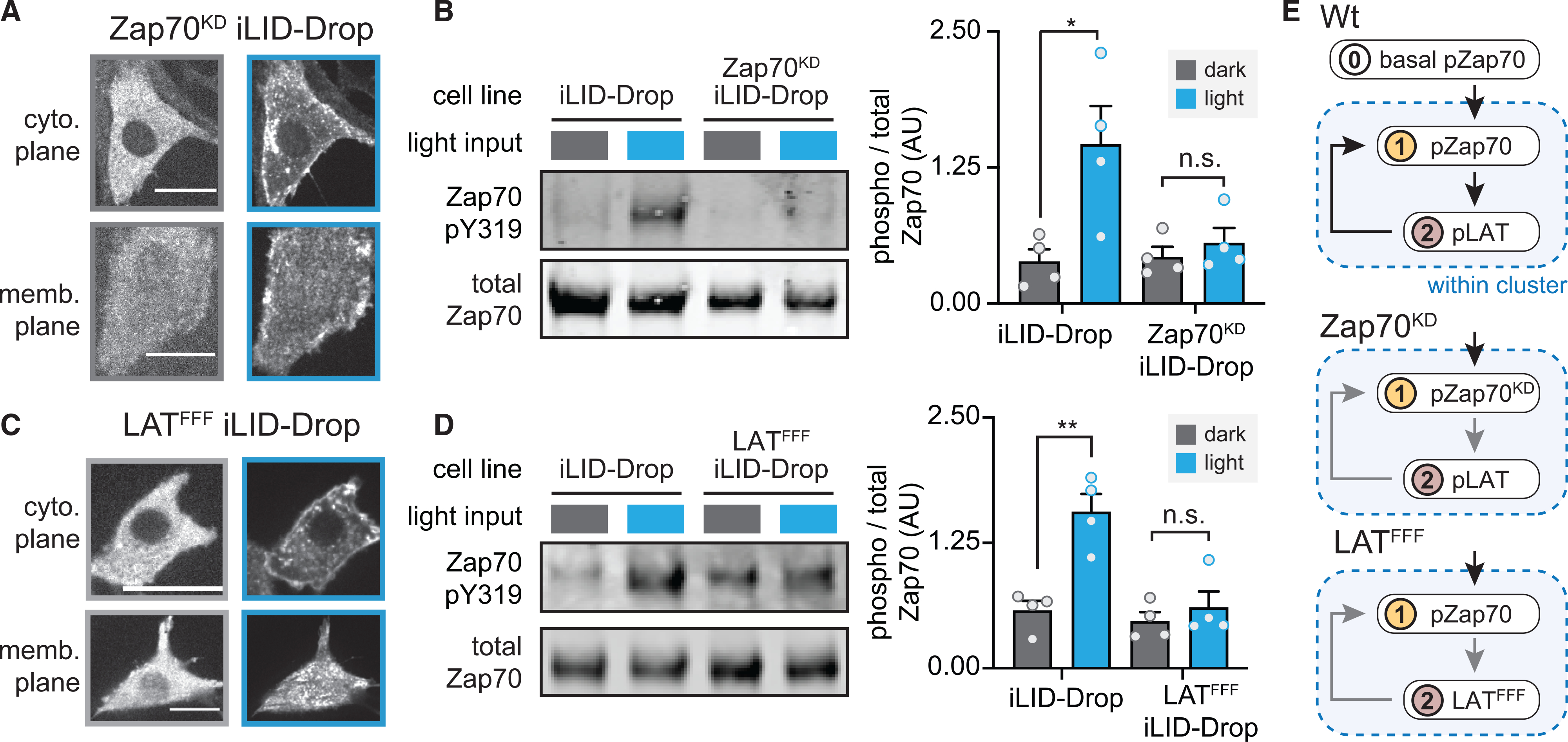
Positive feedback links Zap70 kinase activity and LAT substrate phosphorylation with further Zap70 activation (A) Images of kinase-dead tRFP-Zap70^K369R^ (Zap70^KD^) in iLID-Drop cells. Images show cytosolic and membrane planes. Scale bars, 20 μm. (B) Western blot and quantification of pY319-Zap70 for the indicated cell lines. (C) Images of tRFP-Zap70 localization in LAT^FFF^ (LAT Y171F, Y191F, Y226F) iLID-Drop cells. (D) Western blot and quantification of pY319-Zap70 in the indicated cell lines. (E) Schematic of positive feedback loop between LAT and Zap70 in the iLID-Drop clusters. No increase in pZap70 is observed in Zap70^KD^ or LAT^FFF^ cells, demonstrating that this upstream event depends on downstream steps. For (A)–(D), cells were incubated in the dark (gray) or after 20 min of light (blue). Graphs display mean ± SEM for all independent biological replicates (points). All statistical comparisons were performed using Student’s t test using all independent biological replicates. *p < 0.05, **p < 0.01, ***p < 0.001, and ****p < 0.0001. See also Figure S3.

What occurs within Zap70:LAT clusters to promote Zap70 phosphorylation? To gain insight into this process, we set out to establish the requirements for clustering-based signaling using LAT and Zap70 mutant variants. We first tested whether Zap70 kinase activity is required by constructing an iLID-Drop variant containing a kinase-dead Zap70 mutant, Zap70^K369R^ (Liaunardy-Jopeace et al., 2017). This kinase-dead variant failed to induce Zap70 phosphorylation, even though illumination still produced membrane-associated Zap70:LAT clusters (Figure S3C), indicating that Zap70 phosphorylation depends on Zap70 kinase activity (Figures 4A and 4B). We also tested whether Zap70 phosphorylation required the presence of LAT as a substrate, using an iLID-Drop variant in which LAT was replaced by a variant (LAT^FFF^) that lacks the tyrosines initially targeted by Zap70 for phosphorylation (Houtman et al., 2006; Su et al., 2016; Zhang et al., 2000). Again, no light-induced increase in Zap70 phosphorylation was observed in LAT^FFF^ iLID-Drop cells, despite light-induced Zap70 membrane localization and clustering (Figures 4C, 4D, and S3C). iLID-Drop variants harboring each single Y-to-F mutation in LAT still robustly triggered downstream signaling (Figure S3D), as has been observed in T cells, suggesting that the requirement is not restricted to any single Tyr residue (Zhang et al., 2000).

Taken together, our data show that only clusters containing catalytically active Zap70 and phosphorylatable LAT can be fully activated. The dependency of an upstream event (Zap70 phosphorylation) on downstream attributes (Zap70 kinase activity and a phosphorylatable LAT substrate) is indicative of a positive feedback loop operating within Zap70:LAT clusters (Figure 4E). This feedback loop may operate as follows: a low amount of basally phosphorylated Zap70 phosphorylates LAT within the cluster, which—through an as-yet-undefined mechanism—triggers additional phosphorylation and activation of Zap70. Fully active Zap70 further phosphorylates LAT, culminating with the activation of downstream signaling pathways.

### SFKs implement feedback linking LAT phosphorylation to Zap70 activation

We next sought to identify the kinase that mediates Zap70 phosphorylation within membrane-associated Zap70:LAT clusters. During T cell activation, Zap70 Tyr319 is phosphorylated by the SFK Lck (Brdicka et al., 2005; Williams et al., 1999). Although NIH 3T3 and HEK293T cells do not normally express Lck, they do possess general purpose SFKs (Src, Yes, and Fyn) that could substitute for Lck in illuminated iLID-Drop cells.

**Figure 5.**
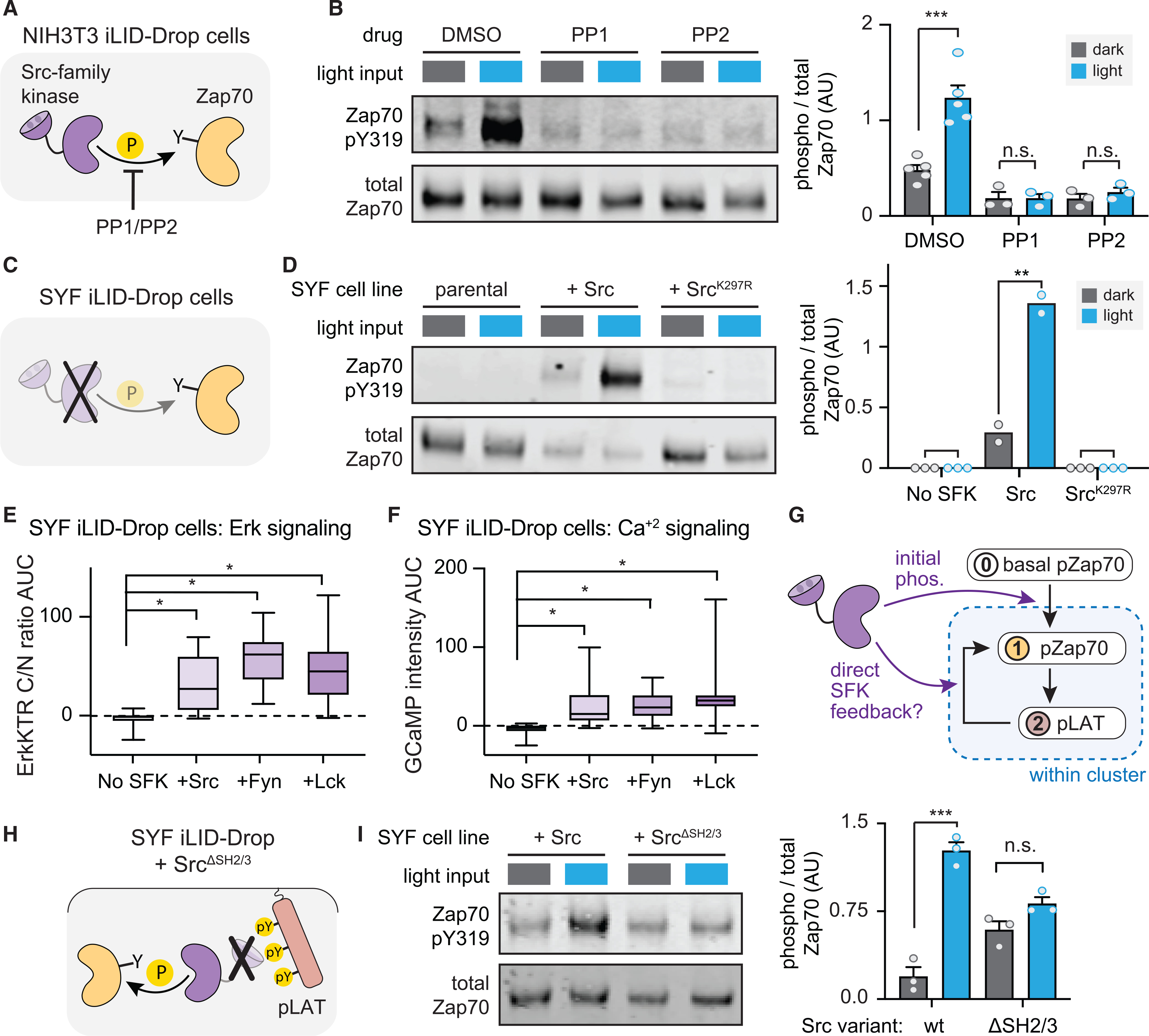
Positive-feedback-driven Zap70 activation depends on SFK activity (A) Schematic SFK inhibitor treatment in iLID-Drop cells. (B) Western blot and quantification of phospho-Zap70 after 20-min treatment with PP1 and PP2 versus DMSO. (C) Schematic of experiments in iLID-Drop SYF cells. (D) Western blot and quantification of pY319-Zap70 in indicated cell lines. (E and F) AUC of ErkKTR-irFP (E) or GCaMP (F) responses for iLID-Drop SYF cells. Boxes represent 25th–75th percentile, center line shows the mean, and whiskers show minimum and maximum values. n ≥ 20 cells from two different experiments. (G) Schematic of potential SFK inputs into the LAT-Zap70 feedback circuit. (H) Schematic of experiments in Src^ΔSH2−3^ iLID-Drop SYF cells. (I) Western blot and quantification of pY319-Zap70 in the indicated SYF cell lines. Bar graphs display mean ± SEM and independent biological replicates (points). All statistical comparisons were performed using Student’s t test using all independent biological replicates. *p < 0.05, **p < 0.01, ***p < 0.001, and ****p < 0.0001. See also Figure S4.

We began by testing whether light-induced Zap70 phosphorylation in NIH 3T3 cells depends on SFK activity using the small-molecule kinase inhibitors PP1 or PP2 to inhibit SFK activity (Figure 5A). Indeed, we observed that these inhibitors eliminated Zap70 Tyr319 phosphorylation in all conditions (Figure 5B). For cleaner control over SFK activity, we next expressed the iLID-Drop system in “SYF” mouse embryonic fibroblasts (MEFs) that were engineered to lack all three ubiquitous SFKs (Src, Yes, and Fyn) (Klinghoffer et al., 1999). As in the PP1/PP2 experiments, we found that clustering-induced Zap70 phosphorylation was completely abolished in SYF fibroblasts regardless of illumination conditions but was restored by expression of Src (Figures 5C and 5D). This restoration required Src kinase activity, as SYF iLID-Drop cells expressing a kinase-dead Src allele (Src^K297R^) also failed to produce Zap70 phosphorylation (Figure 5D).

To further probe the generality of our results, we characterized the dependence of clustering-induced signaling on the identity and expression levels of the SFKs present in our experiments. We first tested whether any of three different SFKs (Src, Fyn, or Lck) were similarly capable of rescued clustering-induced signaling. Indeed, we found that iLID-Drop SYF cells expressing Src, Fyn, or Lck triggered similar levels of Erk and calcium signaling (Figures 5E and 5F). Second, we quantified Src expression levels in Src-transduced SYF cells by western blotting, finding that these cells expressed ~100-fold-higher levels of Src than NIH 3T3 cells (Figures S4A and S4B). Despite these differences in expression level, downstream pathway activation still robustly depended on LAT-Zap70 cluster formation, as iLID-Only expressing SYF-MEFs failed to mount a signaling response (Figure S4C). Taken together, our data demonstrate that SFK activity is essential for Zap70 activation and LAT phosphorylation in non-T cells, and this effect appears to be robust across different SFK family members and a wide range of their expression levels.

Based on our data and classic studies of Zap70 activation (Williams et al., 1999; Yan et al., 2013), we envisioned two potential roles for SFKs. First, leaky SFK activity may be required to provide an initial basal level of Zap70 phosphorylation, which was observed in dark-incubated Zap70-expressing cells throughout our study (Figures 3C and 5D). This leaky activity may be a prerequisite for initial Zap70 phosphorylation of LAT, which might be amplified by SFK-independent positive feedback to generate full phosphorylation of Zap70. Second, SFKs may also directly participate in positive feedback by binding to phospho-LAT and then phosphorylating nearby Zap70 molecules, further increasing Zap70 activity and LAT phosphorylation (Figure 5G). This second possibility is supported by structural studies of Src activation: Src contains an SH2 domain that can lock it in an auto-inhibited conformation until it binds to pTyr residues, which both tethers Src to a potential substrate and increases its activity (Boggon and Eck, 2004). The binding of an SFK’s SH2 domain to LAT’s pTyrs, possibly strengthened further by binding between the SFK’s SH3 domain and a proline-rich motif on LAT (Lo et al., 2018), could thus trigger recruitment and local activation of SFKs within the cluster, driving further Zap70 and LAT phosphorylation in a positive feedback loop (Aten et al., 2013; Feng and Cooper, 2009).

To separate these two potential functions of SFKs, we set out to introduce a “feedback-disconnected” Src variant that could still drive basal Zap70 phosphorylation but not participate in positive feedback within Zap70:LAT clusters. To do so, we deleted the SH2 and SH3 domains from our previously made TagBFP-tagged Src (Src^ΔSH2/3^-BFP). This Src variant should lack all autoinhibitory interactions and so exhibit high activity, supporting basal Zap70 phosphorylation. However, it should also lack any protein association domains for recruitment to phospho-LAT, thereby blocking any potential role in cluster-localized positive feedback (Figure 5H). We engineered iLID-Drop SYF fibroblasts to express either Src^ΔSH2/3^-BFP or Src-BFP at levels that resulted in basal Zap70 phosphorylation in the dark and tested both cell lines for an increase in Zap70 phosphorylation upon light stimulation. As before, we found that Zap70 phosphorylation increased upon light stimulation in Src-BFP iLID-Drop cells (Figure 5I). This effect was dramatically reduced in Src^ΔSH2ΔSH3^-BFP iLID-Drop cells, which showed similar levels of phosphorylation in both dark and light and failed to attain the high levels of Zap70 phosphorylation observed in light-stimulated Src-BFP cells (Figure 5I). We did observe a slight, but not statistically significant, increase in pZap70 in light-treated Src^ΔSH2ΔSH3^-BFP iLID-Drop cells. Any remaining change in Zap70 activation might reflect the combined effect of any clustering-sensitive effects other than SFK-mediated positive feedback, such as the potential exclusion of cellular phosphatases from Zap70:LAT clusters (Su et al., 2016; Zhang, 2002).

Our data can be readily interpreted in the context of a simple conceptual model: a cluster-localized positive feedback loop involving Zap70, LAT, and an SFK. Basally phosphorylated Zap70 leads to weak LAT phosphorylation, followed by SFK recruitment and activation through SH2-mediated binding to phosphorylated LAT (pLAT). The SFK then further phosphorylates nearby Zap70 proteins within the cluster, completing the feedback loop. Strikingly, the system appears to function as a high-fidelity sensor of clustering state, with all-or-none signaling differences observed between clustered and un-clustered LAT, even when the identity or expression level of the SFK is varied.

### A mathematical model recapitulates signaling through cluster-localized positive feedback

The presence of feedback can make it extremely difficult to intuit the behavior of a biochemical network, even when such a system consists of only three components. We thus wondered whether we could recapitulate our experimental observations—including the responses observed from clusters, dimers, and various mutant proteins—using a minimal mathematical model of the three-component signaling circuit. We reasoned that such a model could be tested for its sufficiency to recapitulate our experimental observations and to explore additional scenarios for further insights into the Zap70/LAT/SFK module.

**Figure 6.**
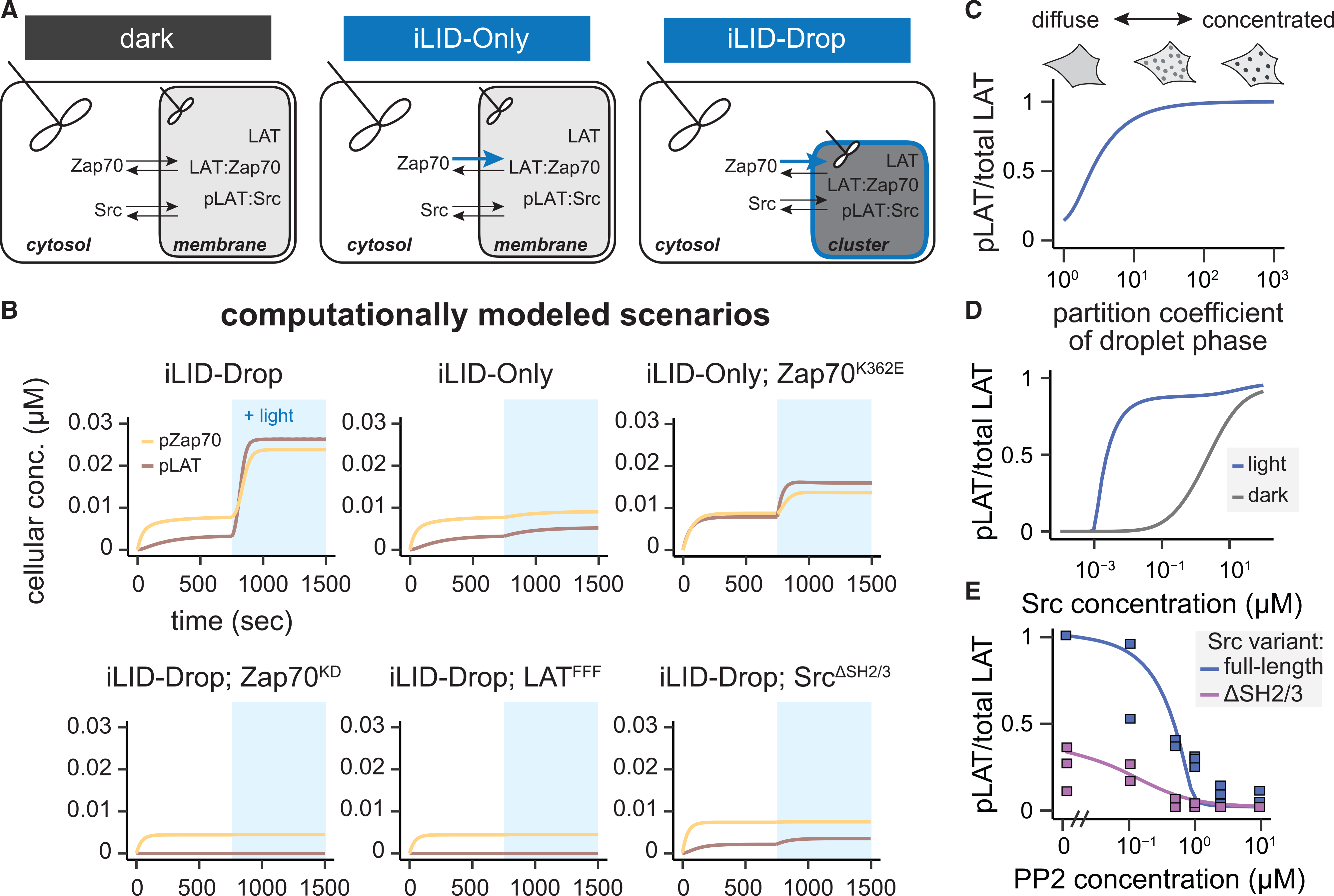
A mathematical model of positive feedback recapitulates sensitivity to clustering state and robustness to SFK concentration (A) Schematic of the model. In the dark, the modeled cell consists of two well-mixed compartments (indicated by the stir propellors) representing the cytosol and the membrane. In iLID-Only simulations, light stimulation recruits Zap70 to the membrane, while in in iLID-Drop, this effect is combined with a 10-fold drop in the membrane compartment volume. (B) Simulated cellular concentrations of pZap70 (yellow) and pLAT (brown) following light stimulation (blue) in six different experimental scenarios. (C and D) The modeled ratio of pLAT to total LAT is shown as a function of LAT’s partition coefficient on the membrane (C) and the cellular concentration of Src (D). Curves represent iLID-Drop simulations in dark (gray) and light (blue) conditions. (E) iLID-Drop simulations (lines) and experimental replicates (points) for the ratio of pLAT to total LAT in the presence of the Src inhibitor PP2 in SYF iLID-Drop fibroblasts expressing either wt Src (blue) or Src^ΔSH2−3^ (purple). See also Figure S5 and Tables S1–S2.

Our model contains three proteins (LAT, Zap70, and Src) that can occupy two cellular compartments: a cytosolic compartment containing free Zap70 and Src; and a membrane-localized compartment containing LAT, bound Zap70:LAT, and bound Src:p-LAT (Figure 6A). To model optogenetic stimulation, we first assume that Zap70 has an increased propensity to phosphorylate LAT when the two proteins are tethered by light-induced iLID-SspB dimerization. Second, we model the light-induced formation of LAT clusters as a simple decrease in their available volume, thus leading LAT and any LAT-bound proteins to become proportionally concentrated in the cluster. Overall, the model includes two binding interactions (light-induced Zap70:LAT binding via iLID-SspB dimerization [Guntas et al., 2015] and Src:p-LAT binding through its SH2 domain [Felder et al., 1993]) and three phosphorylation reactions (weak Zap70 phosphorylation by free Src; strong Zap70 phosphorylation by Src:p-LAT complexes; and LAT phosphorylation by p-Zap70). Finally, we assume constitutive, first-order dephosphorylation of LAT and Zap70. Where possible, we inferred model parameters from experimental measurements of the relevant proteins (Methods details; Tables S1 and S2).

We first tested whether this model recapitulated key findings from our experiments. We simulated the model in six experimental scenarios: iLID-Drop, iLID-Only, iLID-Only Zap70^K362E^, iLID-Drop Zap70^KD^, iLID-Drop LAT^FFF^, and iLID-Drop Src^ΔSH2ΔSH3^ cells. In each case, light stimulation was assumed to trigger a 100-fold decrease in the iLID-SspB dissociation constant and a 10-fold increase in LAT-optoDroplet concentration (in the iLID-Drop case only); all other parameters were held constant. We observed strong light-induced phosphorylation of LAT and Zap70 in the iLID-Drop but not iLID-Only scenario, with similar kinetics and fold-change in phosphorylation as in our experiments (Figure 6B). The model also matched results from key mutations, showing minimal activity in simulations lacking Zap70/Src kinase activity or phosphorylatable LAT. Our model also requires Src-mediated positive feedback: a Src^ΔSH2ΔSH3^ allele that cannot bind phospho-LAT results in an intermediate level of phosphorylation regardless of illumination conditions (Figure 6B). The model thus confirms that a clustering-based positive feedback loop is sufficient to quantitatively explain our data across a wide range of experimental conditions.

We next used the model to interrogate the striking combination of sensitivity and robustness revealed by our experiments. It appears that signaling depends sensitively on whether LAT is clustered (Figures 3B and 3C) and yet appears to be robust to a ~100-fold variation in SFK expression (Figure S4A). What degree of LAT clustering is required to trigger a potent signaling response, and over what range of Src concentrations might the circuit function? To address these questions, we first modeled LAT phosphorylation in iLID-Drop cells while varying the degree of light-induced clustering (Figure 6C). We observed that signaling increased with the degree of Zap70:LAT clustering, plateauing to a maximum as LAT was concentrated approximately 10-fold above its initial value, well within the range of observed values for protein condensates *in vitro* and in cells (Banani et al., 2016; Bracha et al., 2018). In contrast, we observed strong clustering-induced signaling even as Src levels were varied across at least two orders of magnitude (Figure 6D). This robustness to Src concentration absolutely required positive feedback, as simulating the feedback-disconnected Src^ΔSH2ΔSH3^ allele revealed a gradual increase in LAT and Zap70 phosphorylation that failed to discriminate between clustered and unclustered conditions (Figures S5A and S5B). The model also exhibited a large difference between light and dark signaling even at high cellular Src concentrations where overall Src enrichment in the clusters was low (Figure S5C). Because Src is activated by its interactions with LAT, even a small degree of enrichment could give rise to a large difference between activated Src within the cluster and autoinhibited Src in the cytosol.

As a final probe of the model, we set out to test a prediction in a context not yet measured experimentally: how the signaling module responds to titrating Src activity, not just concentration. We simulated a titration of the small-molecule inhibitor PP2 for both wild-type Src and feedback-disconnected Src^ΔSH2ΔSH3^ and then compared to corresponding experimental results. Once again, we found that model and experiment agreed closely, revealing that wild-type Src elicited higher levels of LAT phosphorylation—and signaled effectively across a broader range of PP2 concentrations—than its feedback-disconnected counterpart (Figure 6E). Taken together, our computational modeling results confirm that the Zap70-LAT-Src positive feedback circuit can indeed act as a sensitive sensor of protein clustering while being robust to variation in other cellular parameters (e.g., the concentration or activity state of Src).

### Optogenetic LAT clustering in Jurkat T cells triggers Ca^2+^ signaling

**Figure 7.**
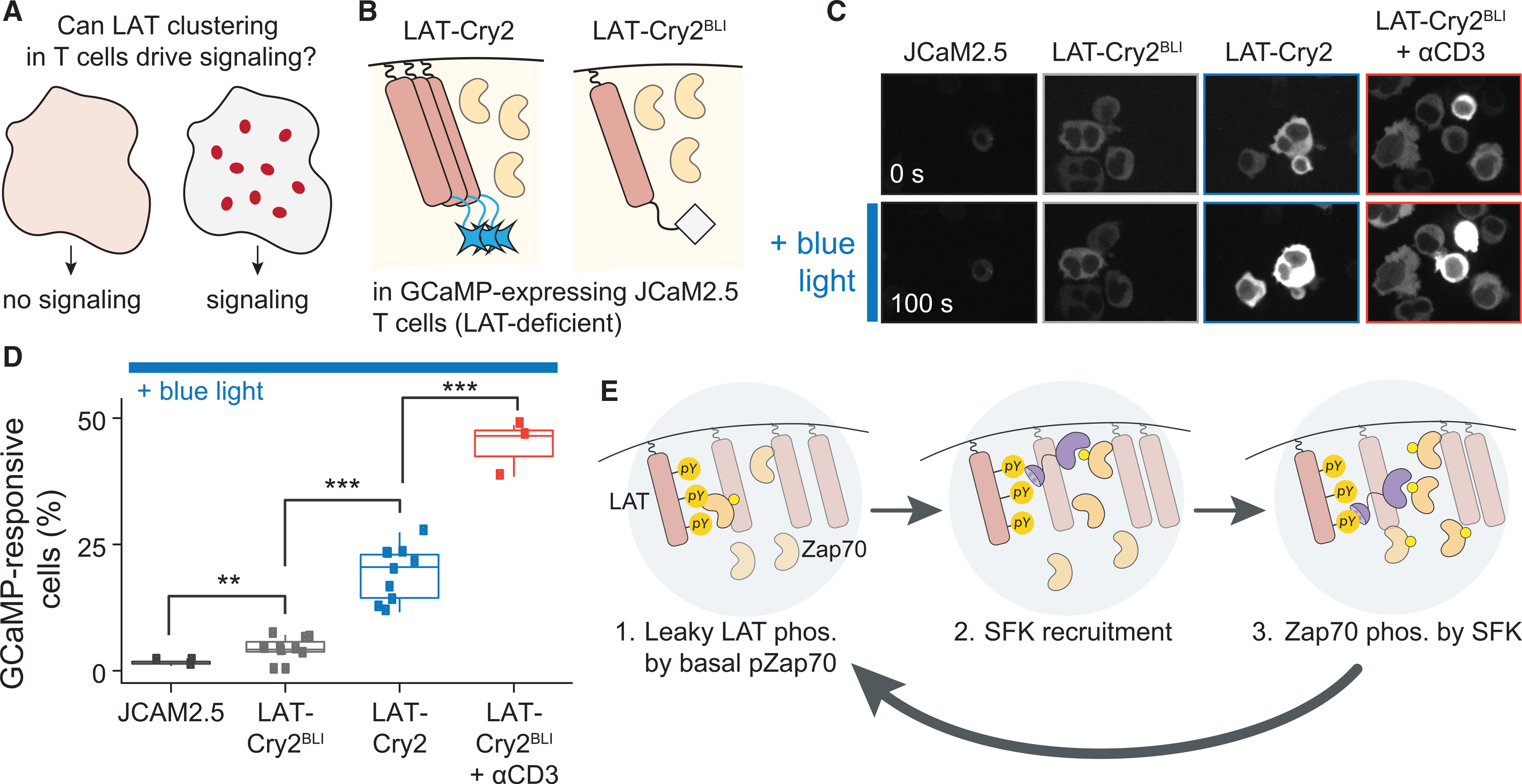
Light-induced LAT-Cry2 clustering triggers calcium signaling in Jurkat T cells (A) Schematic of central hypothesis: LAT clustering triggers positive feedback, leading to a T cell signaling response. (B) LAT-deficient JCAM2.5 Jurkat T cells were transduced with the GCaMP biosensor and either LAT-Cry2 or a light-insensitive variant (LAT-Cry2^BLI^). (C) Representative images for JCaM2.5 GCaMP cells transduced with nothing, LAT-Cry2BLI, or LAT-Cry2. Dark (top), illuminated (bottom), and anti-CD3 antibody (red) stimulation conditions are shown. (D) Quantification of (C) showing the fraction of cells exhibiting a sustained increase in GCaMP fluorescence after stimulation. Each data point represents at least 50 cells from independent experiments. Boxes represent median ± quartiles. **p < 0.01 and ***p < 0.001 as derived from Student’s t test. (E) Schematic of cluster-localized positive feedback among LAT, SFKs, and Zap70. A low amount of basally phosphorylated Zap70 performs some leaky phosphorylation of LAT. Phosphorylated LAT then recruits and activates SFKs through SH2-pTyr and SH3-proline rich motif interactions. Finally, active SFKs phosphorylate and activate additional Zap70 molecules within the cluster, completing the feedback loop. See also Figure S6 and Video S6.

Throughout this study, we have dissected the Zap70-LAT circuit in fibroblasts, far from these proteins’ natural context in T cell signaling. Reconstituting this circuit in an orthogonal cell type had many advantages: it allowed us to manipulate variants of LAT and Zap70 in isolation, without interference from endogenous LAT and Zap70 or other T-cell-specific activators (e.g., Lck, the TCR) or inhibitors (e.g., the CD45 phosphatase) that might alter their localization or activity. Nevertheless, the data presented thus far raise a key question: Can similar optogenetic manipulations reveal a functional role for LAT clusters in T cells (Figure 7A)?

We relied on two crucial simplifications to apply our minimal optogenetic approach to LAT clustering in a native T cell context. First, it was previously observed that Zap70 is already localized near the T cell membrane prior to antigen stimulation (Huby et al., 1997; van Oers et al., 1994), suggesting that LAT clusters might be able to functionally interact with Zap70 in T cells even without additional synthetic recruitment of Zap70. This hypothesis was further strengthened by computational simulations where we observed that LAT clustering can trigger at least partial activation even without direct Zap70 recruitment, provided that a basal pool of p-Zap70 is initially present to initiate positive feedback (Figure S5D). Second, a LAT-deficient Jurkat T cell line (JCAM2.5) was previously established. JCAM2.5 cells are unable to trigger downstream signaling in response to receptor-level stimulation, underscoring LAT’s essentiality for this process and suggesting that any optogenetic LAT variants that we introduce would be the sole source of this required protein for T cell signal transduction, enabling us to test specifically for signaling differences between clustered and diffuse LAT variants.

Our strategy for controlling LAT clustering in T cells relied on a fusion of LAT to the Cry2 optogenetic clustering domain. Cry2 fusion has been successfully used to control clustering of other TCR signaling components (e.g., CD3ζ) (Ma et al., 2020), and we found LAT-Cry2 to be well tolerated when stably expressed in T cells, in contrast to other optogenetic tool variants that we and others have found to be difficult to maintain in stably expressing immune cell lines (Prof. Orion Weiner, UCSF, personal communication). We introduced either LAT-FusionRed-Cry2 (LAT-FR-Cry2) or a variant harboring the light-insensitive D387A mutation (LAT-FR-Cry2^BLI^) into GCaMP-expressing JCAM2.5 cells and sorted similar expression levels of each line (Figure 7B). We then performed optogenetic stimulation and GCaMP imaging in each cell line every 5 s for 3 min in the presence or absence of an anti-CD3 crosslinking antibody (Figures 7C and 7D; Video S6; see Figure S6 for all conditions).

We found that GCaMP fluorescence remained low under all conditions in parental JCAM2.5 cells, consistent with LAT’s essential role in T cell signaling (Figures 7D and S6). LAT-Cry2^BLI^-expressing cells exhibited sporadic bursts of GCaMP fluorescence without a notable change over time while illuminated, consistent with a low light-insensitive baseline of calcium signaling. In contrast, a proportion of LAT-Cry2 cells exhibited a rapid and sustained increase in GCaMP fluorescence upon illumination. Quantifying the proportion of cells with sustained GCaMP responses revealed that light-induced LAT clustering provoked a comparable response to that obtained by anti-CD3 stimulation the same LAT-Cry2 cells (Figure S6) but a weaker response than TCR-level stimulation of LAT-Cry2^BLI^ cells (Figure 7D). Taken together, our data demonstrate that acute, light-induced LAT clustering is indeed sufficient to trigger at least a partial T cell signaling response, suggesting a functional role for signaling protein clusters in their native cellular context.

## DISCUSSION

Protein phase separation and clustering has been proposed to play a role in a wide variety of cellular functions. But in many cases, it remains possible that phase separation is a consequence, not a cause, of signaling pathway activity. Discriminating between these possibilities is especially challenging because so many signaling proteins engage in weak, multivalent binding interactions, such as the binding between SH2 domains and pTyr residues, that would be predicted to be essential for pathway activity regardless of whether the constituent proteins are clustered. In this study, we set out to determine whether the clustering of two T cell signaling proteins, the kinase Zap70 and its substrate LAT, plays a functional role in modulating downstream signaling. Indeed, we found that LAT clusters in T cells and Zap70:LAT clusters in fibroblasts were sufficient to activate canonical downstream pathways, whereas a similar number of Zap70:LAT heterodimers was not. Studies in knockout cell lines and with mutant proteins further revealed the mechanism of cluster-specific signaling: a three-component feedback loop where SFKs bind to phosphorylated LAT, leading to further Zap70 activation (Figure 7E).

One major question in cell signaling has been how to identify the minimal set of protein components that are required for a particular cellular outcome. We propose that additional insights can be gained from testing not just which molecular components must be present in the cell, but also whether they must be present in the context of a certain biophysical state (e.g., within a protein cluster or condensate). For example, previous work demonstrated that in Jurkat T cells, other T-cell-specific proteins such as SLP-76 and GADS are required for downstream signaling (Lugassy et al., 2015; Yablonski et al., 1998); yet we observe that fibroblasts expressing neither SLP-76 nor GADS can activate downstream pathways in response to Zap70:LAT clustering. It may be that those adaptor proteins are essential for nucleating signaling clusters, a function that is provided here by our optogenetic systems. Separating the creation of a biophysical compartment from signal propagation within it could be of great utility for clarifying the essential functions of components within a signaling pathway.

Cells employ biochemical networks to sense a diverse array of upstream inputs, including extracellular ligands, misfolded proteins, and small molecules. Our study defines a three-component signaling circuit that appears to function as a “cluster detector.” Both experiments and computational modeling reveal that the Zap70-LAT-Src circuit responds strongly to the formation of membrane-localized clusters but not lower-order molecular complexes. Moreover, the system appears to function robustly as other parameters are varied (e.g., the cellular concentration or activity of SFKs). We anticipate that variations of this biochemical circuit may find application in diverse contexts, from biosensors to report on the presence of specific condensates (Khan et al., 2018) to synthetic biology studies aiming to engineer signaling circuits using designer membraneless organelles (Chiesa et al., 2020; Reinkemeier et al., 2019; Zhao et al., 2019).

While our study provides experimental and computational evidence for clustering-based positive feedback, many questions remain about its role in the native context: Zap70 phosphorylation and downstream signaling during normal antigen encounter by T cells. Although Zap70 is initially phosphorylated by the TCR-associated kinase Lck, within minutes, LAT and Zap70 form clusters that are spatially distinct from the TCR (Pageon et al., 2016). One role for localized positive feedback may be to trigger sustained signaling from these receptorless LAT:Zap70 clusters. It is also important to note that Zap70 phosphorylation can reach high levels in stimulated T cells that lack LAT clusters, suggesting that LAT-Zap70 positive feedback is dispensable at least in the context of a maximal receptor-level stimulus (Bilal and Houtman, 2015; Houtman et al., 2006; Kortum et al., 2013). However, Zap70 also clusters with many other Tyr-rich substrates, including the TCR itself, and analogous positive feedback may also operate within those clusters.

Recent work has revealed that LAT and Zap70 form condensates in a signaling-dependent manner (Su et al., 2016). Our T cell data provide a clue in the converse direction: LAT clustering is sufficient to initiate at least a partial T cell signaling response. This result is important because it establishes a causal link between LAT molecular organization and downstream signaling in T cells. Nevertheless, it is only a first step. Our T cell results are compatible with multiple detailed biochemical mechanisms, including the originally proposed mechanism of CD45 phosphatase exclusion (Su et al., 2016) and the LAT-Zap70-SFK feedback loop identified here, either of which may act individually or in combination. Further studies that separate these potential cluster-sensing mechanisms in T cells could help evaluate their relative importance in shaping cellular responses. Our results must also be considered in light of the observation that endogenous LAT clusters have been observed in resting T cells without triggering activation (Williamson et al., 2011). Perhaps the difference lies in adaptation to a quiescent state after prolonged clustering-induced activation, as has been observed in the context of activating Zap70 mutants or long-term receptor stimulation (Graef et al., 1997). Alternatively, it may be that additional suppressive factors co-occupy stable LAT clusters. The complexity of the native system suggests that much work remains to be done to understand the myriad roles played by protein clustering during T cell activation. Optogenetic reconstitution presents one possible route to separating these effects, one cluster at a time.

## STAR★METHODS

### RESOURCE AVAILABILITY

#### Lead contact

Further information and requests for resources and reagents should be directed to and will be fulfilled by the lead contact, Jared Toettcher (toettcher@princeton.edu).

#### Materials availability

There are no restrictions on material availability. Plasmids are available from Addgene (www.addgene.org/Jared_Toettcher), and all cell lines produced in this study will be made available upon request.

#### Data and code availability

There are no restrictions on data availability. All data generated or analyzed during this study are included in this published article and its supplementary files or are available upon request. MATLAB code for simulating the computational model is available on the laboratory GitHub page (https://github.com/toettchlab).

### EXPERIMENTAL MODEL AND SUBJECT DETAILS

#### Cell culture

NIH 3T3 as well as Src^−/−^, Yes^−/−^, and Fyn^−/−^ (SYF) mouse embryonic fibroblasts (MEFs) were grown in DMEM supplemented with 10% FBS, 1% L-Glutamine, and Pen/Strep. Cells were maintained on Thermo Scientific Nunc Cell Culture Treated Flasks with Filter Caps and grown at 37°C with 5% CO_2_. These same conditions were used for Lenti-X-293T cells shown in Figure S2. JCAM 2.5 Jurkat cells were grown in RPMI medium supplemented with 10% FBS, 1% L-Glutamine, and Pen/Strep and grown in the same conditions as noted above. Cell lines were obtained from repositories (ATCC) or from the labs that generated the cell lines (for JCAM2.5 Jurkat cells) and were not independently authenticated.

### METHOD DETAILS

#### Plasmids

All plasmids were constructed using InFusion cloning (Clontech) to ligate in a PCR product to a pHR vector that was opened using either backbone PCR.

#### LAT-FR-Cry2 constructs

To create LAT-FR-Cry2, we linearized the myristoylated optoDrop plasmid used in Dine et al. (2018) and replaced the myrostoylation tag and the FUS^N^ domain with full length LAT, obtained LAT from its human ORFeome plasmid (ORFeome Collaboration, 2016). We then conducted site-directed mutagenesis on Cry2 to make the D387A mutations to make the blue light insensitive version.

#### iLID-Drop and iLID-Only constructs

To create iLID-Drop (pHR-RFP-SspB-Zap70-P2A-LAT- iLID-FUS^N^-Cry2) we start with the iLID-SspB SosCat plasmid from Goglia et al. (2020). We removed SOScat and replaced it with Zap70 from its pDONR plasmid. Then we removed the CAAX tag and replaced with FUS^N^-Cry2 from myristoylated optoDrop Plasmid used in Dine et al. (2018). Finally, we linearized the plasmid via backbone PCR to insert LAT from its human ORFeome plasmid (ORFeome Collaboration, 2016) between the P2A and iLID sequences in our construct.

We conducted site-directed mutagenesis on Cry2 to make the D387A mutations for the iLID- Only construct. Site-directed mutagenesis was also used to make constitutively active or Kinase Dead Zap70 dimers as seen in Figures 3 and 4. Site directed mutagenesis was also to make the point mutants for the experiments displayed in Figure S3. For LAT-FFF iLID-Drop construct (Figures 4C and 4D) we used LAT FFF from Su et al., 2016 (Addgene # 78517), instead of WT LAT.

#### Reporter plasmids

We used pHR-ErkKTR-irFP to monitor activity as we had done previously in Dine et al. (2018). We used GCaMP6f to monitor calcium activity by linearizing a pHR vector and inserting GCaMP6f from Addgene plasmid # 10837 (Toettcher et al., 2011).

#### SFK plasmids

We performed backbone PCR to linearize the ErkKTR-BFP plasmid from Goglia et al. (2020) and replaced the ErkKTR with a Src-family kinase (SFK) from its respective pDONR plasmid (Src = Addgene # 23934, Fyn = Addgene # 82211 and Lck = Addgene # 82305). Site-directed mutagenesis was then used to create each of the Src variants studied in Figure 5. To make Src^ΔSH2ΔSH3^-BFP, we removed the sequence coding for amino acids 83–535 in the original pHR-Src-BFP vector and replaced it with an insert with the sequence coding for amino acids 248–535.

#### Lentivirus production and transduction

Lentivirus was produced as per the protocol we described previously^6^. Briefly, Lenti-X 293T cells were plated in a 6-well plate at 20%–30% confluency and co-transfected with the appropriate pHR expression plasmid and lentiviral packaging plasmids (pMD2.G and p8.91 – gifts from the Trono lab) using Fugene HD transfection reagent. Viral supernatants were collected 48–52 hr after transfection and passed through a 0.45 mm filter.

NIH 3T3, SYF-MEFs and Lenti-X 293T cells to be infected with lentivirus were plated in a 6 well dish at 20% – 40% confluency. 500 μl of filtered virus were added to the cells as was 50 μl of 1 M HEPES and 2 μl of 5 μg/ ml polybrene. Cells were then grown up and plated in T75 Nunc Flasks for cell sorting via FACS Aria as described previously (Goglia et al., 2020).

For viral infection of JCAM 2.5 cells, 48 hours before infection, cells were diluted to a density of 1.25 × 10^5^ cells/ml. At the time of infection 1 mL of cells were plated in a 6 well dish and 1 mL of freshly filtered virus was added along with HEPES and polybrene at the concentrations listed above. 24 hours later cells were diluted into 8 additional ml of RPMI media and grown up until reaching a concentration of ~5 × 10^5^ cells/ml in 20ml of RPMI media and then sorting with the FACS Aria as above.

#### Cell preparation for imaging

For imaging experiments involving NIH 3T3 and SYF cell lines, cells were plated on black-walled, 0.17 mm glass-bottomed 96 well plates (*In Vitro* Scientific). Prior to cell plating, glass was pretreated with a solution of 10 μg/mL fibronectin in phosphate buffer saline (PBS) for 5 – 60 min. NIH 3T3 and SYF MEFs were allowed to adhere for at least 4 hours in our supplemented DMEM. Cells were then switched to starvation media (DMEM + 20 μM HEPES) (Toettcher et al., 2013) for 2 hours before imaging. Just prior to imaging 50 μL of mineral oil was added to the top of each well to stop evaporation (Toettcher et al., 2011).

For imaging JCAM 2.5 cells, the same glass-bottomed 96 well plates were pre-treated with CellTak (Corning) according to the manufacturer’s instructions. 2 × 10^4^ cells resting in RPMI medium lacking FBS were then added to the wells and allowed to adhere for 2 hours before imaging and mineral oil was added to the top of each well immediately prior to imaging as above.

#### Time-lapse microscopy

Cells were maintained at 37°C with 5% CO_2_ for the duration of all imaging experiments. Confocal microscopy was performed on a Nikon Eclipse Ti microscope with a Prior linear motorized stage, a Yokogawa CSU-X1 spinning disk, an Agilent laser line module containing 405, 488, 561 and 650 nm lasers, an iXon DU897 EMCCD camera, and 20X air or 40X and 60X oil immersion objective lens. Note that for tRFP-LAT imaging, all images were auto-scaled independently before and after optogenetic stimulation. This auto-scaling was essential because we found that overall tRFP brightness increased by approximately two-fold as a consequence of blue light illumination, leading to an overall brighter signal in light conditions.

Due to the fast off-time of our optogenetic constructs, we were only able to image one region on our microscope for each experiment. Thus, for every NIH 3T3 and SYF cell line experiment, we imaged the ErkKTR with the 650 nm laser, Zap70 localization with the 561 nm laser and GcAMP6f with the 488 nm laser. We acquired these images every 15 s for 15 min or for the images in Figure 1 and Videos S1 and S2 every 5 s for 5 min. Between each acquisition, we used a 450 nm LED light source (XCite XLED1) delivered through a Polygon400 digital micromirror device (DMD; Mightex Systems) to deliver a constant input of blue light. We set the blue light LED to half its maximal intensity but allowed all the light to pass through the mirrors (no dithering) so as to provide a strong enough light input for each position imaged.

We used a 488 nm laser to deliver blue light for optogenetic stimulation to the Jurkat cell lines and to image GcAMP6f in those cells. We acquired images every 5 s for 3 minutes using a 40X Nikon oil immersion objective. For each field of view, a 3 by 3 array of adjacent frames were stitched together.

#### Cell lysis and western blotting

To prepare cells for stimulation and lysis 24 hours prior to experiment cells plated into Nunc 6-well dishes at 30% - 40% confluency. The day of experiment cells were checked to be between 60% - 70% confluency. The media was then removed and replaced with 2 mL of starvation media for 2 hr. Cells were either kept in the dark or stimulated with blue light.

Blue light was delivered via custom-printed boards containing small 450 nm LED bulbs. These boards were placed on top of foil wrapped boxes that were placed in our 37°C incubator. The 6-well dishes containing the cells were then added to the boxes and the blue light board was placed on top of the boards so as to directly stimulate only our cells of interest. Blue light was applied at a constant 5V for 20 min.

Following the 20 min stimulation the media was quickly removed and cells were placed on ice and treated with 120 μl of RPPA lysis buffer (1% Triton X-100, 50 mM HEPES buffer, 150 mM NaCl, 1.5 mM MgCl_2_, 1 mM EGTA, 100 mM NaF, 10 mM sodium pyrophosphate, 1 mM Na_3_VO_4_, 10% glycerol, freshly-prepared protease/phosphatase inhibitors). Cell scrapers were then used to collect the cells and each lysate was transferred to Eppendorf tubes on ice. Lysates were then spun down at 4°C for 10 min at 13,300 x g. Supernatants were transferred to new tubes where 40 μl 4X NuPAGE LDS Sample Buffer (Thermo Fisher) was added to each, and samples were boiled at 98°C for 5 min.

Samples were then run on a gel for western blotting done as described previously in Goglia et al., 2020^1^. Primary antibodies used in this study are listed on the table above and all were used at 1:1,000 dilution, except for anti-GAPDH, which was used at 1:2500. Fluorescent secondary antibodies, 800CW goat anti-rabbit and IRDye 680RD goat anti-mouse were purchased for Li-Cor and used at a 1:10,000 dilution. Blots were then imaged on a Li-Cor Odyssey CLx imaging system.

#### Drug additions

To inhibit SFK activity PP1 and PP2 were reconstituted at a concentration 10 mM in DMSO and kept at −20°C. Immediately prior to cell stimulation with blue light (or darkness) PP1 and PP2 were diluted a total of 1,000 fold in starvation media (for a final concentration of 10 μM) and added to cells to acutely inhibit SFK activity. To stimulate Jurkats, anti-CD3 was added to the wells to a final, saturating, concentration of 2 μg/mL.

### QUANTIFICATION AND STATISTICAL ANALYSIS

#### Image analysis

##### Measuring KTR and GCaMP values for NIH 3T3 and SYF cells

Image analysis was performed in ImageJ. For KTR analysis equivalent nuclear or cytoplasmic regions were tracked over time by hand annotation. We then measured the mean fluorescent intensity in each annotated region for every time point. We then background subtracted every measured and plotted the cytoplasmic/nuclear ratio for each time point. Graphs showing mean and SEM values for each time point were made with GraphPad Prism, and Area Under the Curve (AUC) was calculated by subtracting the initial cytosolic to nuclear ratio from the value at each time point and summing up all those differences for each of the 61 time points. The box and whisker plots show the 25 – 75^th^ percentile values for individual cells within the population with the whiskers showing min and max values and the line in the middle of the box showing the mean.

For GCaMP analysis, a small area was drawn in a randomly chosen cytoplasmic region of each cell. Mean fluorescent intensities were measured and background subtracted as above. Values were then normalized to the minimum value found in each cell’s individual trace. Graphs were generated as was done for ErkKTR. AUC was calculated as was done for the ErkKTR. All details regarding the statistical testing including n and p values as well as the types of statistical test used can be found in the figure legends for each data figure.

##### Measuring GCaMP values for Jurkat cells

To measure GCaMP values in our Jurkat experiments, a CellProfiler pipeline was used to identify the cells in each field of view, track the cells for each frame of a 3 min time course, and calculate the average pixel intensity for each cell. Only cells that CellProfiler was able to successfully track throughout the duration of the time course were included in further analysis. For each cell, the GCaMP response was normalized to the minimum value in the trace. Cells were then categorized based upon whether or not it displayed a sustained GCaMP response. To be considered as responding, the cell must have had a pixel intensity above a noise threshold. Additionally, to distinguish sustained responses from transient blinking, the average intensity must have remained above its half-maximal value for a minimum of 30 s during the 3 min time course to be considered a sustained GCaMP response. The fraction of responding cells was then calculated for each field of view. These fractions were then plotted in Rstudio using ggplot and compared with a Student’s t test for statistical testing. More details on the statistical tests including n and p values can be found in the legends to Figures 7 and S6.

#### Statistical analysis of western blots

Images of Western Blots from the Li-Cor Odyssey CLx imaging system were analyzed using imageJ software to calculate pixel intensities for all bands of interest. Pixel intensities from phospho-species antibodies were divided by the corresponding total species value as indicated in each figure. For the blot measuring LAT or Src expression levels in Figures S1B and S4A respectively, the band intensities in the 680 channel (anti-LAT or anti-Src) was normalized to the 800 channel (anti-GAPDH). Plotting and statistical analysis for all blots was performed using GraphPad Prism. Non-paired Student’s t tests were used to compare dark and light conditions for each different cell line or drug treatment. All further details regarding the statistical testing including n and p values can be found in the figure legends for each data figure.

#### Computational model

Our computational model consists of three species: LAT, Src, and Zap70 that appear in two cellular compartments: the membrane/cluster compartment and the cytosol. LAT resides in the membrane compartment, and Src and Zap70 reside in the cytosol and can diffuse freely into the membrane compartment. We used mass action kinetics to describe phosphorylation of Zap70 and LAT in the dark state and after illumination with blue light under several scenarios. We also compared the steady state extent of phosphorylation given by our model to the experimentally measured values (Table S1) and parameters given in Table S2.

To simulate the formation of LAT clusters upon illumination, we decreased the volume of the membrane compartment by a factor, **K**, such that:

$$V_{\mathrm{clust}\;}=\frac{V_{\mathrm{mem}\;}}{K}\;[LAT]=\text{K}{\left[LAT\right]}_{0}$$

Additionally, Zap70 binds to LAT by an iLID/SspB interaction upon illumination. We assumed that diffusion and binding is much faster than phosphorylation and can therefore be approximated to be at equilibrium and that the cytosolic concentration of Zap70 remains constant. Furthermore, we assumed that Zap70 will also freely diffuse into the clusters, therefore:

$$[\mathrm{Zap}70]=[LAT]\frac{{\left[Zap70\right]}_{0}K_{A}^{iLID/SspB}}{1+{\left[Zap70\right]}_{0}K_{A}^{iLID/SspB}}+{\left[Zap70\right]}_{0}$$

Src can bind to phosphorylated LAT through a SH2/pY interaction and this binding releases autoinhibition. As before, we approximated binding to be at equilibrium and assumed that the cytosolic concentration of Src remains constant. Src will also freely diffuse into the cluster, remaining in an inhibited state, therefore:

$${\left[\mathrm{Src}\right]}_{a}=[LAT]\frac{{\left[\mathrm{Src}\right]}_{0}K_{A}^{pY/SH2}}{1+{\left[\mathrm{Src}\right]}_{0}K_{A}^{pY/SH2}}\;{\left[\mathrm{Src}\right]}_{i}={\left[\mathrm{Src}\right]}_{0}$$

We modeled Zap70 phosphorylation by Src using Michaelis-Menten kinetics. In the cluster, Zap70 will be rapidly phosphorylated by active Src and slowly phosphorylated by autoinhibited, inactive Src. In the cytosol, Zap70 will be phosphorylated by inactive Src. Furthermore, we assumed that Zap70 undergoes constitutive dephosphorylation following first order kinetics:

$$\frac{d}{dt}{\left[\mathrm{Zap}70\right]}_{p}=k_{\mathrm{cat}}^{{\mathrm{Src}}_{a}}{\left[\mathrm{Src}\right]}_{a}\frac{{\left[\mathrm{Zap}70\right]}_{n}}{K_{M}^{{\mathrm{Src}}_{a}}+{\left[Zap70\right]}_{n}}+k_{\mathrm{cat}}^{{\mathrm{Src}}_{i}}{\left[\mathrm{Src}\right]}_{i}\frac{{\left[Zap70\right]}_{n}}{K_{M}^{{\mathrm{Src}}_{i}}+{\left[Zap70\right]}_{n}}-k_{n}^{Zap70}{\left[Zap70\right]}_{p}\;\frac{d}{dt}{\left[\mathrm{Zap}70\right]}_{p,cyt}=k_{\mathrm{cat}}^{{\mathrm{Src}}_{i}}{\left[\mathrm{Src}\right]}_{i}\frac{{\left[\mathrm{Zap}70\right]}_{n,cyt}}{K_{M}^{{\mathrm{Src}}_{i}}+{\left[Zap70\right]}_{n,cyt}}-k_{n}^{Zap70}{\left[Zap70\right]}_{p,cyt}$$

Finally, we modeled phosphorylation of LAT by pZap70. For simplicity, we considered only one phosphorylatable tyrosine on LAT. We allowed LAT to be phosphorylated by two distinct mechanisms: (1) phosphorylation by pZap in the cluster following Michaelis-Menten kinetics, and (2) preferential phosphorylation of LAT by pZap70 that is bound to it by an iLID—SspB interaction following first order kinetics. Additionally, we assumed that LAT undergoes constitutive dephosphorylation following first order kinetics.

LAT that is not bound to Zap70 can only be phosphorylated by the first mechanism, therefore:

$$\frac{d}{dt}{\left[LAT\right]}_{p,\mathrm{free}\;}=k_{\mathrm{cat}}^{pZap70}{\left[\mathrm{Zap}70\right]}_{p}\frac{{\left[LAT\right]}_{n,\mathrm{free}}}{K_{M}^{\mathrm{pZap}70}+{\left[LAT\right]}_{n,\mathrm{free}}}-k_{n}^{LAT}{\left[LAT\right]}_{p,\mathrm{free}}$$

LAT that is bound to Zap70 may be phosphorylated by either phosphorylation mechanism, therefore:

$$\frac{d}{dt}{\left[LAT\right]}_{p,\mathrm{bound}}=k_{\mathrm{cat}}^{pZap70}{\left[Zap70\right]}_{p}\frac{{\left[LAT\right]}_{n,\mathrm{bound}}}{K_{M}^{pZap70}+{\left[LAT\right]}_{n,\mathrm{bound}}}+\;k_{pZap70}{\left[nLAT-pZap70\right]}_{\mathrm{iLID}}-k_{n}^{LAT}{\left[LAT\right]}_{p,\mathrm{bound}}$$

In the preceding equation, [*nLAT*−*pZap*70]_*iLID*_ is the pool of iLID:SspB bound LAT:Zap70 complexes that consist of non-phosphorylated LAT and phosphorylated Zap70, given by

$${\left[nLAT-pZap70\right]}_{\mathrm{iLID}}={\left[LAT\right]}_{n,\mathrm{bound}}\frac{{\left[\mathrm{Zap}70\right]}_{p}}{\left[\mathrm{Zap}70\right]}$$

PP2 was modeled as a non-competitive inhibitor, therefore, the catalytic rate constants for active and inactive Src were scaled by:

$$k_{cat}^{\mathrm{Src},PP2}=k_{cat}^{\mathrm{Src}}\frac{K_{l}}{K_{l}+[\mathrm{PP}2]}$$

To model different experimental regimes, parameters and reactions were altered in the model as follows. iLID-Drop: The base scenario, with all equations and parameters as indicated in this section and using parameters in Table S2. iLID-Only: We modeled light-insensitive Cry2 by setting **K** to 1 in both dark and lit states. Zap70-K362E: We modeled weak constitutive Zap70 activity by allowing both phosphorylated and non-phosphorylated Zap70 to phosphorylate LAT with a lower rate constant and higher Michaelis constant than wild-type p-Zap70. Zap70KD: We modeled kinase-dead Zap70 by setting the rate constant for LAT phosphorylation by Zap70 to 0. LATFFF: We modeled non-phosphorylatable LAT by disallowing Src binding to pLAT in the model, and also setting the catalytic rate constant for LAT phosphorylation by Zap70 to 0. SrcΔSH2ΔSH3: We modeled this Src mutant by disallowing Src binding to pLAT in the model, increasing the rate constant for phosphorylation of Zap70 by free Src and reducing the overall cellular concentration of Src, to account for the reduced expression observed for mutant Src-expressing cells.

All simulations were performed in MATLAB version R2020a, using ode23 to solve the differential equations. Graphs generated from the model were plotted in R Studio version 1.1.456.

## Supplementary Material

1

2

3

4

5

6

7

8

## Figures and Tables

**KEY RESOURCES TABLE:** 

REAGENT or RESOURCE	SOURCE	IDENTIFIER

Antibodies		

Phospho-Y319 Zap70 rabbit antibody	Cell Signaling Technologies	Cat # 2717S
		RRID: AB_2218658
Total Zap70 mouse antibody	Cell Signaling Technologies	Cat # 2709S
		RRID: AB_2257531
Phospho Y191 LAT rabbit antibody	Cell Signaling Technologies	Cat # 3584s
		RRID: AB_2157728
Phospho Y132 LAT rabbit antibody	Invitrogen	Cat # 44-244
		RRID: AB_2533608
Total Lat mouse monoclonal antibody	Invitrogen	Cat# 14-9967-82
		RRID: AB_1235011
Total GAPDH rabbit antibody	Cell Signaling Technologies	CST: 2118s
		RRID: AB_561053
Phospho Erk 1/2 rabbit antibody	Cell Signaling Technologies	Cat # 4370s
		RRID: AB_2315112
Total Erk 1/2 mouse antibody	Cell Signaling Technologies	Cat # 4696s
		RRID: AB_390780
Total Src mouse monoclonal antibody	Cell Signaling Technologies	Cat# 2210s
		RRID: AB_10691385
Anti-CD3 Human monoclonal antibody	Invitrogen	Cat# 14-0037-82
		RRID: AB_467057

Bacterial and virus strains		

Stellar Chemically Competent Cells	Clontech Laboratories	Cat # 636763

Chemicals, peptides, and recombinant proteins		

CloneAmp HiFi PCR Polymerase	Clontech Laboratories	Cat # 639298
DMSO	Sigma-Aldrich	Cat# D8418
Fugene HD	Promega	Cat# E2311
inFusion HD cloning kit	ClonTech Laboratories	Cat # 638911
PP1-Calbiochem	Millipore-Sigma	Cat # 567809
PP2-Calbiochem	Millipore-Sigma	Cat # 529573
PrimeSTAR GXL DNA Polymerase	Clontech Laboratories	Cat # R050B

Experimental models: Cell lines		

NIH 3T3 cells	American Type Culture Collection (ATCC)	Cat # CRL-1658
Lenti-X 293 cells	Clontech Laboratories	Cat # 632180
SYF-Mouse Embryonic Fibroblasts	ATCC	Cat # CRL-2459
JCAM 2.5 Jurkat Cells	Gift from Dr. Arthur Weiss (Finco et al., 1998)	RRID: CVCL_DR61

Recombinant DNA		

pHR-LAT-FR-Cry2	This paper	N/A
pHR-LAT-FR-Cry2BLI	This paper	N/A
pHR-iLID-Drop	This paper	Addgene #171038
pHR-iLID-Only	This paper	Addgene #171037
pHR-iLID-Drop Zap70^Y319F^	This paper	N/A
pHR-iLID-Only Zap70^K362E^	This paper	N/A
pHR-iLID-Drop Zap70^K369R^	This paper	N/A
pHR-iLID-Drop LAT-FFF	This paper	Addgene #171032
pHR-iLID-Drop LAT^Y171F^	This paper	N/A
pHR-iLID-Drop LAT^Y191F^	This paper	N/A
pHR-iLID-Drop LAT^Y226F^	This paper	N/A
pHR-Src-BFP	This paper	Addgene #171036
pHR-Fyn-BFP	This paper	Addgene #171035
pHR-Lck-BFP	This paper	Addgene #171034
pHR Src^K297R^-BFP	This paper	N/A
pHRSrc *Δ*SH2-SH3	This paper	Addgene #171033
pHR-GCaMP6f	Obtained from Addgene	Addgene plasmid (#10837)
pHR-ErkKTR-irFP	Dine et al., 2018	Addgene # 11510
pCMV-dR8.91 lenti helper plasmid	Gift from Prof. Didier Trono, EPFL	Addgene #12263
pMD2.G lenti helper plasmid	Gift from the Prof. Didier Trono, EPFL	Addgene #12259

Software and algorithms		

Fiji	Schindelin et al., 2012	http://fiji.sc; RRID: SCR_00228
GraphPad Prism	GraphPad Inc.	https://www.graphpad.com
R Studio 1.1.456	RStudio	https://rstudio.com; RRID:SCR_000432
MATLAB R2020a	MathWorks	https://mathworks.com/products/MATLAB/RRID; SCR_001622
CellProfiler	Carpenter et al., 2006	https://cellprofiler.org; RRID: SCR_007358
Github repository for this paper’s MATLAB code	This paper	https://github.com/toettchlab

## Highlights

- Protein clusters versus dimers in cells can be studied with optogenetic tools
- Clusters, but not dimers, of the kinase Zap70 and its substrate LAT trigger signaling
- Positive feedback connects phospho-LAT to Zap70 activation via Src-family kinases
- Optogenetic LAT clustering is sufficient for a calcium response in T cells

## References

[R1] Abraham RT, and Weiss A (2004). Jurkat T cells and development of the T-cell receptor signalling paradigm. Nat. Rev. Immunol 4, 301–308. 15057788 10.1038/nri1330

[R2] Alberti S, Gladfelter A, and Mittag T (2019). Considerations and Challenges in Studying Liquid-Liquid Phase Separation and Biomolecular Condensates. Cell 176, 419–434. 30682370 10.1016/j.cell.2018.12.035 PMC6445271

[R3] Aten TM, Redmond MM, Weaver SO, Love CC, Joy RM, Lapp AS, Rivera OD, Hinkle KL, and Ballif BA (2013). Tyrosine phosphorylation of the orphan receptor ESDN/DCBLD2 serves as a scaffold for the signaling adaptor CrkL. FEBS Lett 587, 2313–2318. 23770091 10.1016/j.febslet.2013.05.064 PMC3759512

[R4] Banani SF, Rice AM, Peeples WB, Lin Y, Jain S, Parker R, and Rosen MK (2016). Compositional Control of Phase-Separated Cellular Bodies. Cell 166, 651–663. 27374333 10.1016/j.cell.2016.06.010 PMC4967043

[R5] Banjade S, and Rosen MK (2014). Phase transitions of multivalent proteins can promote clustering of membrane receptors. eLife 3, e04123. 10.7554/eLife.04123 PMC4238058 25321392

[R6] Bilal MY, and Houtman JCD (2015). GRB2 Nucleates T Cell Receptor-Mediated LAT Clusters That Control PLC-γ1 Activation and Cytokine Production. Front. Immunol 6, 141. 25870599 10.3389/fimmu.2015.00141 PMC4378308

[R7] Boggon TJ, and Eck MJ (2004). Structure and regulation of Src family kinases. Oncogene 23, 7918–7927. 15489910 10.1038/sj.onc.1208081

[R8] Bracha D, Walls MT, Wei MT, Zhu L, Kurian M, Avalos JL, Toettcher JE, and Brangwynne CP (2018). Mapping Local and Global Liquid Phase Behavior in Living Cells Using Photo-Oligomerizable Seeds. Cell 175, 1467–1480.e13. 30500534 10.1016/j.cell.2018.10.048 PMC6724719

[R9] Brdicka T, Kadlecek TA, Roose JP, Pastuszak AW, and Weiss A (2005). Intramolecular regulatory switch in ZAP-70: analogy with receptor tyrosine kinases. Mol. Cell. Biol 25, 4924–4933. 15923611 10.1128/MCB.25.12.4924-4933.2005 PMC1140569

[R10] Bugaj LJ, Choksi AT, Mesuda CK, Kane RS, and Schaffer DV (2013). Optogenetic protein clustering and signaling activation in mammalian cells. Nat. Methods 10, 249–252. 23377377 10.1038/nmeth.2360

[R11] Carpenter AE, Jones TR, Lamprecht MR, (2006). CellProfiler: image analysis software for identifying and quantifying cell phenotypes. Genome Biol 7, R100. 10.1186/gb-2006-7-10-r100. 17076895 PMC1794559

[R12] Case LB, Ditlev JA, and Rosen MK (2019). Regulation of Transmembrane Signaling by Phase Separation. Annu. Rev. Biophys 48, 465–494. 30951647 10.1146/annurev-biophys-052118-115534 PMC6771929

[R13] Chiesa G, Kiriakov S, and Khalil AS (2020). Protein assembly systems in natural and synthetic biology. BMC Biol 18, 35. 32216777 10.1186/s12915-020-0751-4 PMC7099826

[R14] Das J, Ho M, Zikherman J, Govern C, Yang M, Weiss A, Chakraborty AK, and Roose JP (2009). Digital signaling and hysteresis characterize ras activation in lymphoid cells. Cell 136, 337–351. 19167334 10.1016/j.cell.2008.11.051 PMC2662698

[R15] Di Bartolo V, Mège D, Germain V, Pelosi M, Dufour E, Michel F, Magistrelli G, Isacchi A, and Acuto O (1999). Tyrosine 319, a newly identified phosphorylation site of ZAP-70, plays a critical role in T cell antigen receptor signaling. J. Biol. Chem 274, 6285–6294. 10037717 10.1074/jbc.274.10.6285

[R16] Dine E, and Toettcher JE (2018). Optogenetic Reconstitution for Determining the Form and Function of Membraneless Organelles. Biochemistry 57, 2432–2436. 29373016 10.1021/acs.biochem.7b01173 PMC5972035

[R17] Dine E, Gil AA, Uribe G, Brangwynne CP, and Toettcher JE (2018). Protein Phase Separation Provides Long-Term Memory of Transient Spatial Stimuli. Cell Syst 6, 655–663.e5. 29859829 10.1016/j.cels.2018.05.002 PMC6023754

[R18] Dong TX, Othy S, Jairaman A, Skupsky J, Zavala A, Parker I, Dynes JL, and Cahalan MD (2017). T-cell calcium dynamics visualized in a ratio-metric tdTomato-GCaMP6f transgenic reporter mouse. eLife 6, e32417. 29239725 10.7554/eLife.32417 PMC5747524

[R19] Dustin ML, and Groves JT (2012). Receptor signaling clusters in the immune synapse. Annu. Rev. Biophys 41, 543–556. 22404679 10.1146/annurev-biophys-042910-155238 PMC4000727

[R20] Felder S, Zhou M, Hu P, Ureña J, Ullrich A, Chaudhuri M, White M, Shoelson SE, and Schlessinger J (1993). SH2 domains exhibit high-affinity binding to tyrosine-phosphorylated peptides yet also exhibit rapid dissociation and exchange. Mol. Cell. Biol 13, 1449–1455. 7680095 10.1128/mcb.13.3.1449-1455.1993 PMC359455

[R21] Feng L, and Cooper JA (2009). Dual functions of Dab1 during brain development. Mol. Cell. Biol 29, 324–332. 18981215 10.1128/MCB.00663-08 PMC2612505

[R22] Finco TS, Kadlecek T, Zhang W, Samelson LE, and Weiss A (1998). LAT is required for TCR-mediated activation of PLCgamma1 and the Ras pathway. Immunity 9, 617–626. 9846483 10.1016/s1074-7613(00)80659-7

[R23] Gammons M, and Bienz M (2018). Multiprotein complexes governing Wnt signal transduction. Curr. Opin. Cell Biol 51, 42–49. 29153704 10.1016/j.ceb.2017.10.008

[R24] Gan W (2009). Binding specificity of SH2 domains: Insight from free energy simulations. Proteins 74, 996–1007. 18767163 10.1002/prot.22209 PMC2635922

[R25] Goglia AG, Wilson MZ, Jena SG, Silbert J, Basta LP, Devenport D, and Toettcher JE (2020). A Live-Cell Screen for Altered Erk Dynamics Reveals Principles of Proliferative Control. Cell Syst 10, 240–253.e6. 32191874 10.1016/j.cels.2020.02.005 PMC7540725

[R26] Graef IA, Holsinger LJ, Diver S, Schreiber SL, and Crabtree GR (1997). Proximity and orientation underlie signaling by the non-receptor tyrosine kinase ZAP70. EMBO J 16, 5618–5628. 9312021 10.1093/emboj/16.18.5618 PMC1170194

[R27] Guntas G, Hallett RA, Zimmerman SP, Williams T, Yumerefendi H, Bear JE, and Kuhlman B (2015). Engineering an improved light-induced dimer (iLID) for controlling the localization and activity of signaling proteins. Proc. Natl. Acad. Sci. USA 112, 112–117. 25535392 10.1073/pnas.1417910112 PMC4291625

[R28] Hope JM, Liu A, Calvin GJ, and Cui B (2020). Construction of Light-Activated Neurotrophin Receptors Using the Improved Light-Induced Dimerizer (iLID). J. Mol. Biol 432, 3739–3748. 32335036 10.1016/j.jmb.2020.04.018 PMC9879133

[R29] Houtman JCD, Houghtling RA, Barda-Saad M, Toda Y, and Samelson LE (2005). Early phosphorylation kinetics of proteins involved in proximal TCR-mediated signaling pathways. J. Immunol 175, 2449–2458. 16081816 10.4049/jimmunol.175.4.2449 PMC1414060

[R30] Houtman JCD, Yamaguchi H, Barda-saad M, Braiman A, Bowden B, Appella E, Schuck P, and Samelson LE (2006). Oligomerization of signaling complexes by the multipoint binding of GRB2 to both LAT and SOS1. Nat. Struct. Mol. Biol 13, 798–805. 16906159 10.1038/nsmb1133

[R31] Huang WYC, Alvarez S, Kondo Y, Lee YK, Chung JK, Lam HYM, Biswas KH, Kuriyan J, and Groves JT (2019). A molecular assembly phase transition and kinetic proofreading modulate Ras activation by SOS. Science 363, 1098–1103. 30846600 10.1126/science.aau5721 PMC6563836

[R32] Huby RDJ, Iwashima M, Weiss A, and Ley SC (1997). ZAP-70 protein tyrosine kinase is constitutively targeted to the T cell cortex independently of its SH2 domains. J. Cell Biol 137, 1639–1649. 9199177 10.1083/jcb.137.7.1639 PMC2137816

[R33] James JR, and Vale RD (2012). Biophysical mechanism of T-cell receptor triggering in a reconstituted system. Nature 487, 64–69. 22763440 10.1038/nature11220 PMC3393772

[R34] Khan T, Kandola TS, Wu J, Venkatesan S, Ketter E, Lange JJ, Rodríguez Gama A, Box A, Unruh JR, Cook M, and Halfmann R (2018). Quantifying Nucleation In Vivo Reveals the Physical Basis of Prion-like Phase Behavior. Mol. Cell 71, 155–168.e7. 29979963 10.1016/j.molcel.2018.06.016 PMC6086602

[R35] Klinghoffer RA, Sachsenmaier C, Cooper JA, and Soriano P (1999). Src family kinases are required for integrin but not PDGFR signal transduction. EMBO J 18, 2459–2471. 10228160 10.1093/emboj/18.9.2459 PMC1171328

[R36] Kortum RL, Balagopalan L, Alexander CP, Garcia J, Pinski JM, Merrill RK, Nguyen PH, Li W, Agarwal I, Akpan IO, (2013). The ability of Sos1 to oligomerize the adaptor protein LAT is separable from its guanine nucleotide exchange activity in vivo. Sci. Signal 6, ra99. 24222714 10.1126/scisignal.2004494 PMC4259567

[R37] Li P, Banjade S, Cheng H-C, Kim S, Chen B, Guo L, Llaguno M, Hollingsworth JV, King DS, Banani SF, (2012). Phase transitions in the assembly of multivalent signalling proteins. Nature 483, 336–340. 22398450 10.1038/nature10879 PMC3343696

[R38] Liang SI, van Lengerich B, Eichel K, Cha M, Patterson DM, Yoon TY, von Zastrow M, Jura N, and Gartner ZJ (2018). Phosphorylated EGFR Dimers Are Not Sufficient to Activate Ras. Cell Rep 22, 2593–2600. 29514089 10.1016/j.celrep.2018.02.031 PMC5916813

[R39] Liaunardy-Jopeace A, Murton BL, Mahesh M, Chin JW, and James JR (2017). Encoding optical control in LCK kinase to quantitatively investigate its activity in live cells. Nat. Struct. Mol. Biol 24, 1155–1163. 29083415 10.1038/nsmb.3492 PMC5736103

[R40] Liu H, Yu X, Li K, Klejnot J, Yang H, Lisiero D, and Lin C (2008). Photoexcited CRY2 interacts with CIB1 to regulate transcription and floral initiation in Arabidopsis. Science 322, 1535–1539. 18988809 10.1126/science.1163927

[R41] Lo W-L, Shah NH, Ahsan N, Horkova V, Stepanek O, Salomon AR, Kuriyan J, and Weiss A (2018). Lck promotes Zap70-dependent LAT phosphorylation by bridging Zap70 to LAT. Nat. Immunol 19, 733–741. 29915297 10.1038/s41590-018-0131-1 PMC6202249

[R42] Lo WL, Shah NH, Rubin SA, Zhang W, Horkova V, Fallahee IR, Stepanek O, Zon LI, Kuriyan J, and Weiss A (2019). Slow phosphorylation of a tyrosine residue in LAT optimizes T cell ligand discrimination. Nat. Immunol 20, 1481–1493. 31611699 10.1038/s41590-019-0502-2 PMC6858552

[R43] Lugassy J, Corso J, Beach D, Petrik T, Oellerich T, Urlaub H, and Yablonski D (2015). Modulation of TCR responsiveness by the Grb2-family adaptor, Gads. Cell. Signal 27, 125–134. 25452106 10.1016/j.cellsig.2014.10.005

[R44] Ma Y, Lim YJ, Benda A, Lou J, Goyette J, and Gaus K (2020). Clustering of the ζ-chain can initiate t cell receptor signaling. Int. J. Mol. Sci 21, 3498. 10.3390/ijms21103498 PMC7279048 32429097

[R45] Mayer BJ, and Yu J (2018). Protein Clusters in Phosphotyrosine Signal Transduction. J. Mol. Biol 430, 4547–4556. 29870724 10.1016/j.jmb.2018.05.040 PMC6195463

[R46] Nakamura H, Lee AA, Afshar AS, Watanabe S, Rho E, Razavi S, Suarez A, Lin YC, Tanigawa M, Huang B, (2018). Intracellular production of hydrogels and synthetic RNA granules by multivalent molecular interactions. Nat. Mater 17, 79–89. 29115293 10.1038/nmat5006 PMC5916848

[R47] Nandagopal N, Santat L, Lebon L, Sprinzak D, Bronner M, and Elowitz M (2018). Dynamic Ligand Discrimination in the Notch Pathway. Cell 172, 869–880.e19. 29398116 10.1016/j.cell.2018.01.002 PMC6414217

[R48] Collaboration ORFeome (2016). The ORFeome Collaboration: a genome-scale human ORF-clone resource. Nat. Methods 13, 191–192. 26914201 10.1038/nmeth.3776

[R49] Pageon SV, Tabarin T, Yamamoto Y, Ma Y, Nicovich PR, Bridgeman JS, Cohnen A, Benzing C, Gao Y, Crowther MD, (2016). Functional role of T-cell receptor nanoclusters in signal initiation and antigen discrimination. Proc. Natl. Acad. Sci. USA 113, E5454–E5463. 27573839 10.1073/pnas.1607436113 PMC5027455

[R50] Pan L, Fu T-M, Zhao W, Zhao L, Chen W, Qiu C, Liu W, Liu Z, Piai A, Fu Q, (2019). Higher-Order Clustering of the Transmembrane Anchor of DR5 Drives Signaling. Cell 176, 1477–1489.e14. 30827683 10.1016/j.cell.2019.02.001 PMC6529188

[R51] Payne G, Shoelson SE, Gish GD, Pawson T, and Walsh CT (1993). Kinetics of p56lck and p60src Src homology 2 domain binding to tyrosine-phosphorylated peptides determined by a competition assay or surface plasmon resonance. Proc. Natl. Acad. Sci. USA 90, 4902–4906. 7685110 10.1073/pnas.90.11.4902 PMC46621

[R52] Regot S, Hughey JJ, Bajar BT, Carrasco S, and Covert MW (2014). High-sensitivity measurements of multiple kinase activities in live single cells. Cell 157, 1724–1734. 24949979 10.1016/j.cell.2014.04.039 PMC4097317

[R53] Reinkemeier CD, Girona GE, and Lemke EA (2019). Designer membraneless organelles enable codon reassignment of selected mRNAs in eukaryotes. Science 363, eaaw2644. 30923194 10.1126/science.aaw2644 PMC7611745

[R54] Shin Y, and Brangwynne CP (2017). Liquid phase condensation in cell physiology and disease. Science 357, eaaf4382. 28935776 10.1126/science.aaf4382

[R55] Schindelin J, Arganda-Carreras I, Frise E, Kaynig V, Longair M, Pietzsch T, Preibisch S, Rueden C, Saalfeld S, Schmid B, Tinevez JY, White DJ, Hartenstein V, Eliceiri K, Tomancak P, and Cardona A (2012). Fiji: An open-source platform for biological-image analysis. Nature Methods 9 (7), 676–682. 10.1038/nmeth.2019. 22743772 PMC3855844

[R56] Shin Y, Berry J, Pannucci N, Haataja MP, Toettcher JE, and Brangwynne CP (2017). Spatiotemporal Control of Intracellular Phase Transitions Using Light-Activated optoDroplets. Cell 168, 159–171.e14. 28041848 10.1016/j.cell.2016.11.054 PMC5562165

[R57] Spencer DM, Wandless TJ, Schreiber SL, and Crabtree GR (1993). Controlling signal transduction with synthetic ligands. Science 262, 1019–1024. 7694365 10.1126/science.7694365

[R58] Su X, Ditlev JA, Hui E, Xing W, Banjade S, Okrut J, King DS, Taunton J, Rosen MK, and Vale RD (2016). Phase separation of signaling molecules promotes T cell receptor signal transduction. Science 352, 595–599. 27056844 10.1126/science.aad9964 PMC4892427

[R59] Taslimi A, Vrana JD, Chen D, Borinskaya S, Mayer BJ, Kennedy MJ, and Tucker CL (2014). An optimized optogenetic clustering tool for probing protein interaction and function. Nat. Commun. 5, 4925. 25233328 10.1038/ncomms5925 PMC4170572

[R60] Toettcher JE, Gong D, Lim WA, and Weiner OD (2011). Light-based feedback for controlling intracellular signaling dynamics. Nat. Methods 8, 837–839. 21909100 10.1038/nmeth.1700 PMC3184382

[R61] Toettcher JE, Weiner OD, and Lim WA (2013). Using optogenetics to interrogate the dynamic control of signal transmission by the Ras/Erk module. Cell 155, 1422–1434. 24315106 10.1016/j.cell.2013.11.004 PMC3925772

[R62] van Oers NSC, Killeen N, and Weiss A (1994). ZAP-70 is constitutively associated with tyrosine-phosphorylated TCR ζ in murine thymocytes and lymph node T cells. Immunity 1, 675–685. 7600293 10.1016/1074-7613(94)90038-8

[R63] Williams BL, Irvin BJ, Sutor SL, Chini CC, Yacyshyn E, Bubeck Wardenburg J, Dalton M, Chan AC, and Abraham RT (1999). Phosphorylation of Tyr319 in ZAP-70 is required for T-cell antigen receptor-dependent phospholipase C-gamma1 and Ras activation. EMBO J 18, 1832–1844. 10202147 10.1093/emboj/18.7.1832 PMC1171269

[R64] Williamson DJ, Owen DM, Rossy J, Magenau A, Wehrmann M, Gooding JJ, and Gaus K (2011). Pre-existing clusters of the adaptor Lat do not participate in early T cell signaling events. Nat. Immunol 12, 655–662. 21642986 10.1038/ni.2049

[R65] Yablonski D, Kuhne MR, Kadlecek T, and Weiss A (1998). Uncoupling of nonreceptor tyrosine kinases from PLC-γ1 in an SLP-76-deficient T cell. Science 281, 413–416. 9665884 10.1126/science.281.5375.413

[R66] Yan Q, Barros T, Visperas PR, Deindl S, Kadlecek TA, Weiss A, and Kuriyan J (2013). Structural basis for activation of ZAP-70 by phosphorylation of the SH2-kinase linker. Mol. Cell. Biol 33, 2188–2201. 23530057 10.1128/MCB.01637-12 PMC3648074

[R67] Zhang ZY (2002). Protein tyrosine phosphatases: structure and function, substrate specificity, and inhibitor development. Annu. Rev. Pharmacol. Toxicol 42, 209–234. 11807171 10.1146/annurev.pharmtox.42.083001.144616

[R68] Zhang W, Trible RP, Zhu M, Liu SK, McGlade CJ, and Samelson LE (2000). Association of Grb2, Gads, and phospholipase C-gamma 1 with phosphorylated LAT tyrosine residues. Effect of LAT tyrosine mutations on T cell angigen receptor-mediated signaling. J. Biol. Chem 275, 23355–23361. 10811803 10.1074/jbc.M000404200

[R69] Zhao EM, Suek N, Wilson MZ, Dine E, Pannucci NL, Gitai Z, Avalos JL, and Toettcher JE (2019). Light-based control of metabolic flux through assembly of synthetic organelles. Nat. Chem. Biol 15, 589–597. 31086330 10.1038/s41589-019-0284-8 PMC6755918

