# Supplementary material for: Positive feedback between the T cell kinase Zap70 and its substrate LAT acts as a clustering-dependent signaling switch
Source: Cell Rep. Author manuscript; Available in PMC 2021 Jul 21. (PMC8292983; doi:10.1016/j.celrep.2021.109280)
Supplement: 1 [file NIHMS1717852-supplement-1.pdf]

Cell Reports, Volume *35*

Supplemental information

Positive feedback between the T cell kinase Zap70 and its substrate LAT acts as a clustering-dependent signaling switch

Elliot Dine, Ellen H. Reed, and Jared E. Toettcher

Supplementary Figures and Legends

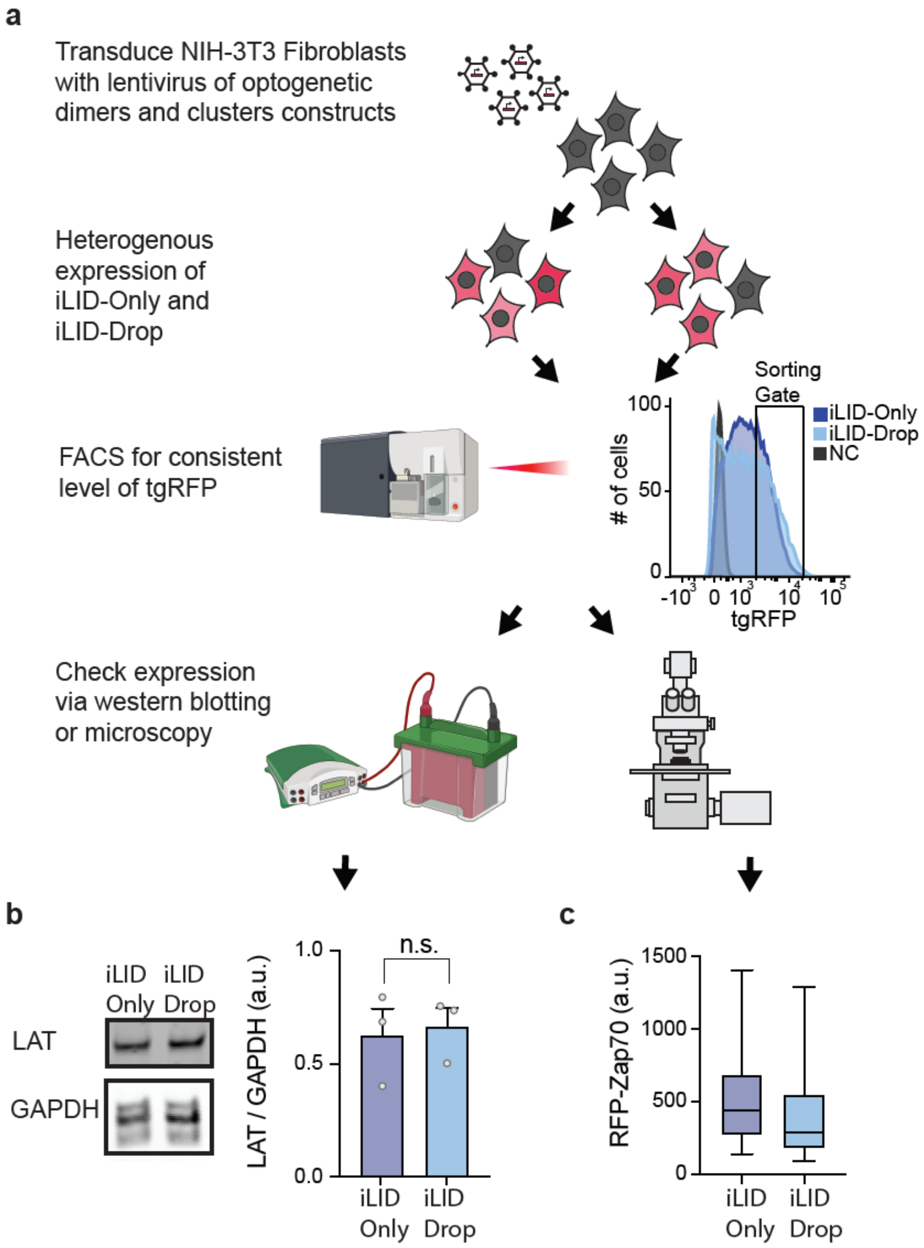

Figure S1: Ensuring that iLID-Only and iLID-Drop optogenetic constructs are expressed at similar levels in NIH-3T3 cell lines, related to Figure 1. (**a**) Flow chart outlining process of creating the iLID-Only and iLID-Drop expressing cell lines used throughout this paper. Plain NIH-3T3 fibroblasts were infected with lentivirus containing constructs depicted in Figure 1B. These stably infected cells were then sorted using a BD systems FACS Aria. The same gate for TagRFP expression were used for both the iLID-Only and iLID-Drop expressing cell lines, as shown. These stable, sorted cell lines were then used for the microscopy and western blotting experiments done in later experiments and to confirm similar levels of expression for both constructs. (**b**) Western blot and quantification of LAT normalized to a loading control (GAPDH) in iLID-Only and iLID-Drop expressing NIH-3T3 cell lines. Graphs display mean, SEM (error bars) and independent biological replicate (dots). $p > 0.05$ from an unpaired Student's T test. (**c**) Box and Whisker plots showing TagRFP (Zap70) fluorescence for iLID-Only and iLID-Drop cells. Boxes represent 25th – 75th percentile with line in the middle representing the mean and whiskers show minimum and maximum. $n = 25$ cells for both conditions.

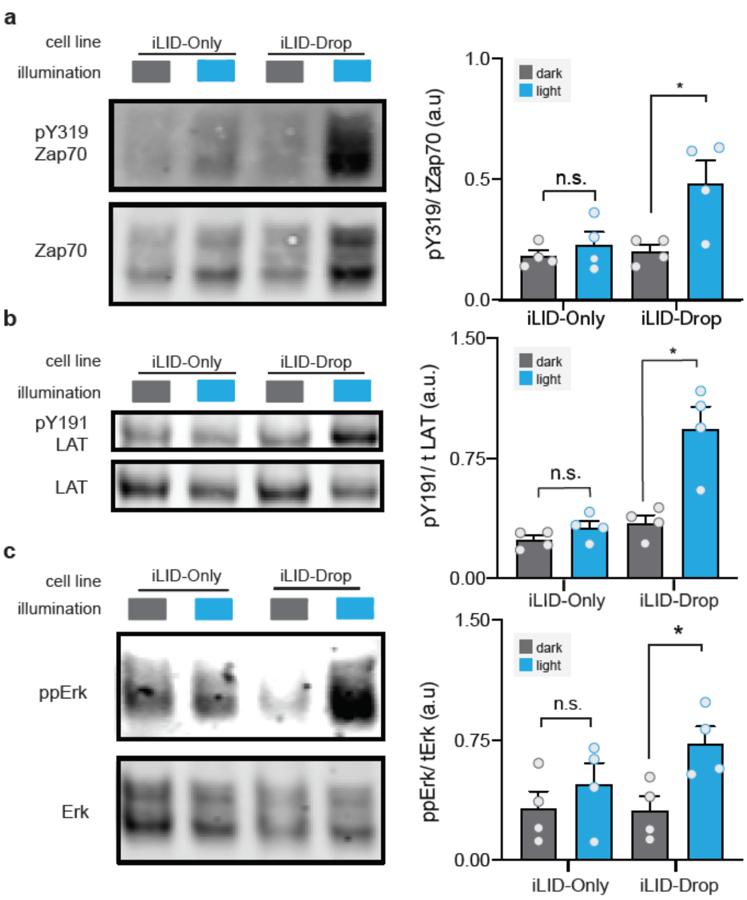

Fig S2: Induction of optogenetic Zap70:LAT clusters leads to successful signaling in HEK-293T cells, related to Figure 3. (**a**) Western blot and quantification for pY319-Zap70 in HEK293T expressing iLID-Only and iLID-Drop cells. For all experiments shown in this figure, cells were either kept in dark (gray bar) or stimulated with blue light for 20 minutes (blue bar). For quantification, dark and light were each compared using the student's t test for all experiments. Graphs display mean, SEM (error bars) and independent biological replicate (dots). * = $p < 0.05$ (**b**) Western blot and quantification for pLAT Y191 in HEK293T expressing iLID-Only and iLID-Drop cells. (**c**) Western blot and quantification of ppERK in HEK293T expressing iLID-Only and iLID-Drop cells.

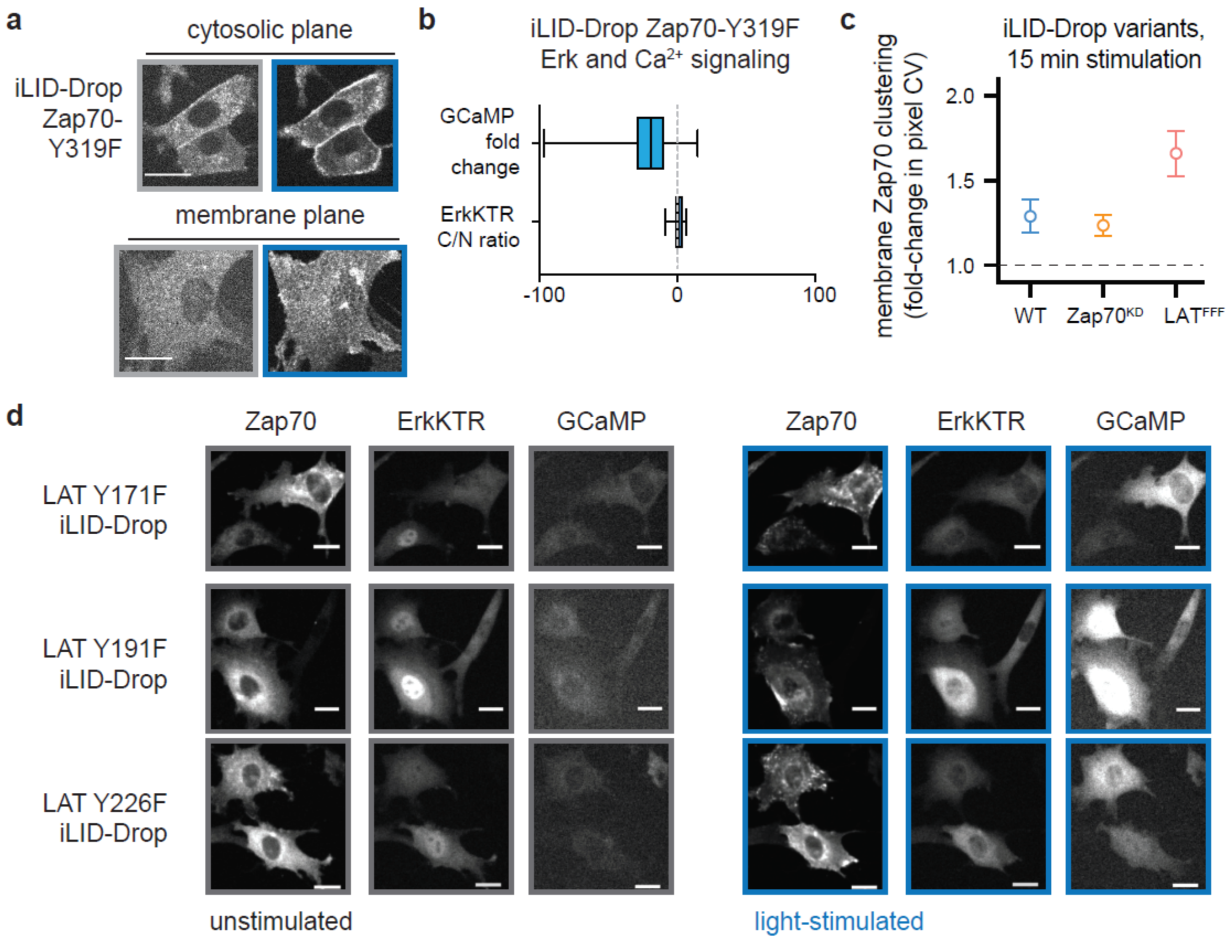

Figure S3: Zap70 phosphorylation is driven by phosphorylation of all three LAT tyrosines, related to Figure 4. (**a**) Images of TagRFP localization in NIH-3T3 cells expressing Zap70^{Y319F} iLID-Drop. Images of Tag-RFP Zap70^{Y319F} were taken at two different planes (cytosolic and membrane) with spinning disk confocal imaging. Gray border indicates images taken prior to blue light illumination, and blue border indicates images taken following 5 minutes of stimulates. Note all Zap70 images underwent contrast enhancement in ImageJ to account for the increase in brightness of TagRFP that occurs when illuminated with blue light. Scale bars = 20 µm. (**b**) Box and Whisker plots showing Area Under the Curve (AUC) for cytoplasmic/nuclear ratios of ErkKTR-irFP and GCaMP fluorescence in Zap70^{Y319F} iLID-Drop expressing NIH-3T3 cells. Boxes represent 25th – 75th percentile with line in the middle representing the mean and whiskers show minimum and maximum. n ≥ 20 cells from 2 different experiments. (**c**) Plot showing average value of the CV of membrane RFP fluorescence following 15 min of blue light stimulation for iLID-Drop Zap70^{KD} and iLID-Drop LAT^{FFF} expressing cells. Values for WT cells are quantified from data shown in Figure 1F. n= 20 cells in each case, plots show mean and SEM. (**d**) Representative images showing localization of Zap70, ErkKTR translocation and GCaMP fluorescence for NIH-3T3 cell lines expressing iLID-Drop with single LAT Y to F mutations. Gray squares around images show cells pre-light stimulation and blue squares around show cells during blue light stimulation (10 min for Zap70 and ErkKTR and 3 min for GCaMP).

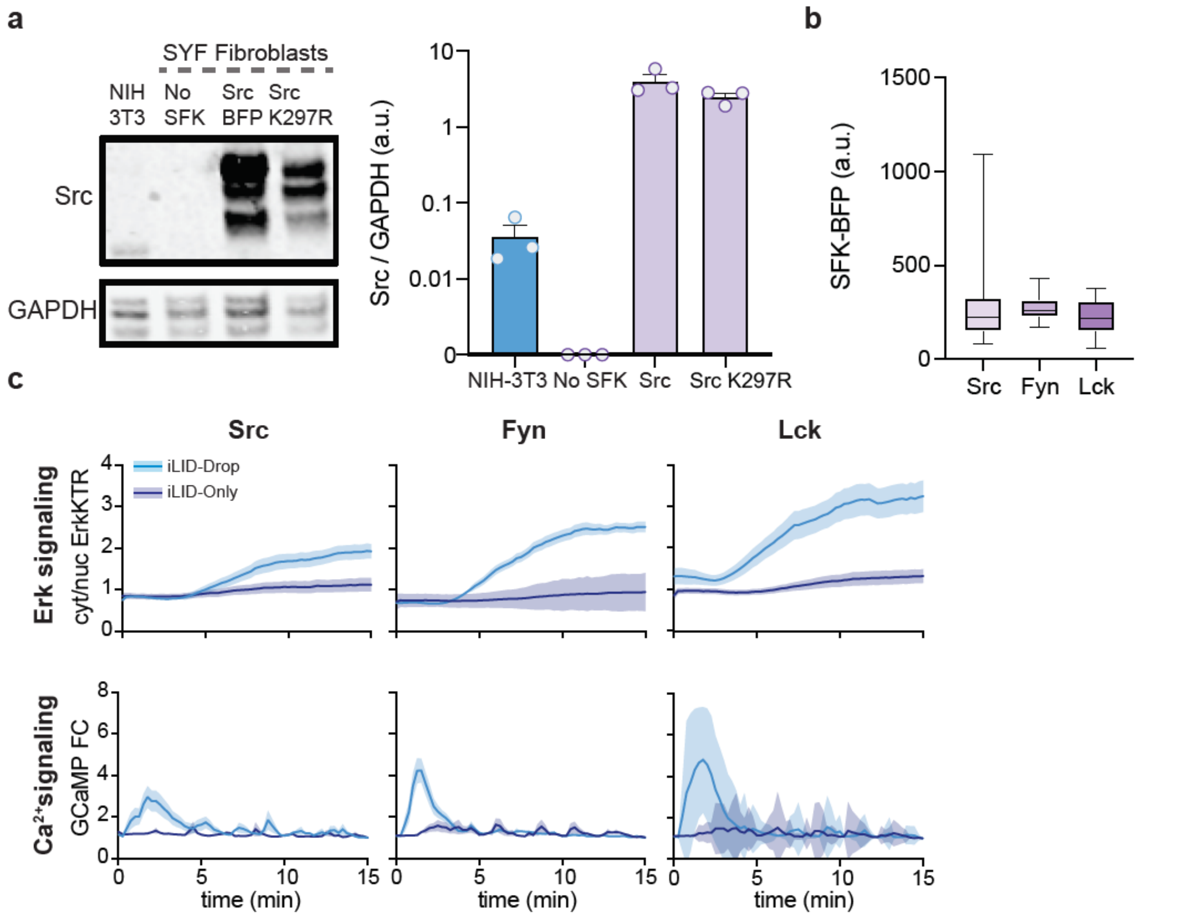

Figure S4: SFK expression levels in engineered SYF-MEF cell lines, related to Figure 5. (**a**) Western blot and quantification of Src normalized to a loading control (GAPDH) in NIH-3T3, SYF, SYF + SRC-BFP and SYF + Src^{K297R}-BFP cell lines. Graphs display mean, SEM (error bars) and independent biological replicate (dots). (**b**) Quantification of fluorescence in 405 nm channel (SFK-BFP) post-FACS sorting for SYF-MEF cell lines different SFKs. n ≥ 20 cells for each cell line. (**c**) Traces of ErkKTR cytoplasmic/nuclear ratio or GCaMP fluorescence for different SYF-MEF cell lines expressing iLID-Only and iLID-Drop constructs. Traces show mean with shaded area representing the SEM. n ≥ 20 cells for every condition.

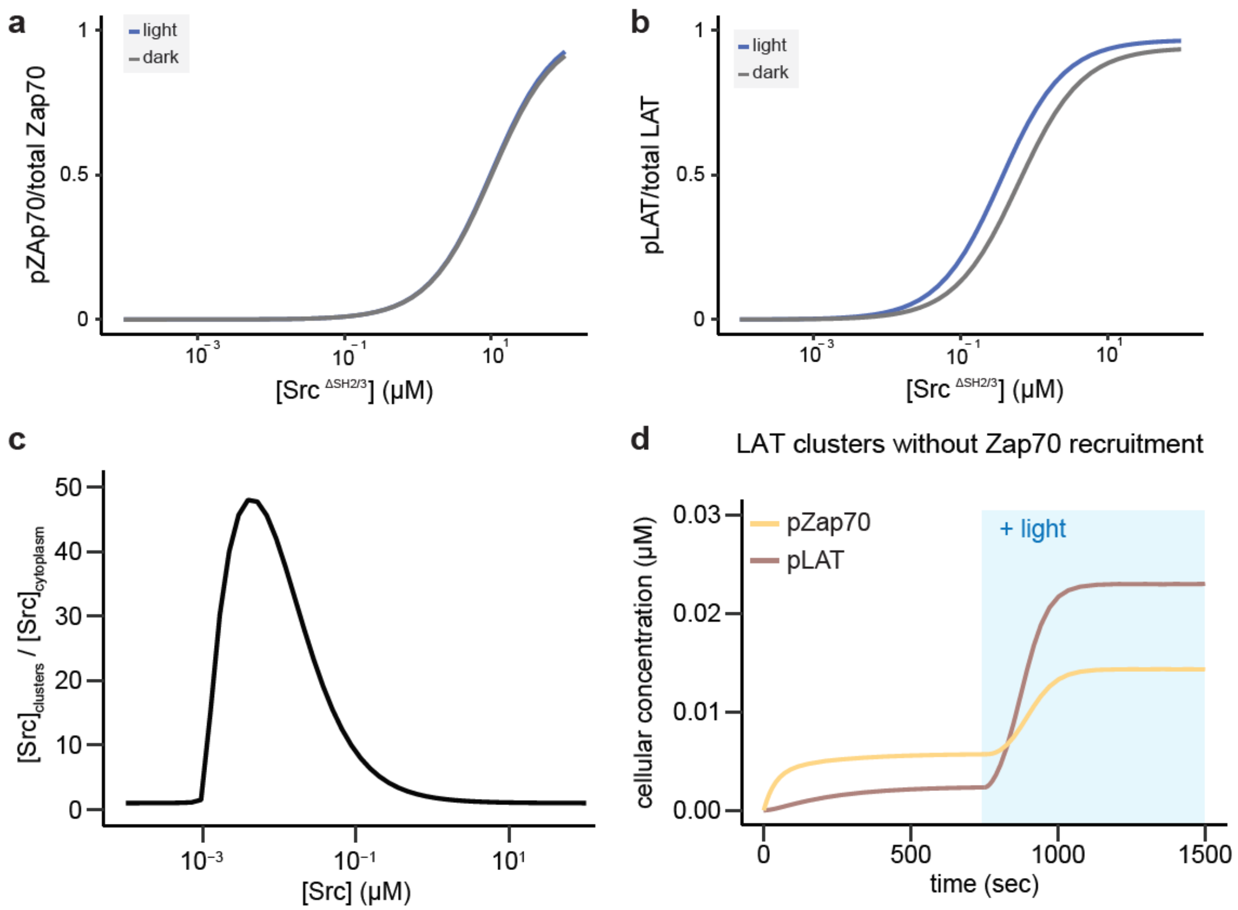

Figure S5: Zap70 and LAT phosphorylation levels display little difference between dark and light stated in simulated cells expressing iLID Drop and Src$^{\Delta SH2\text{-}3}$ related to Figure 6. (**a**) Ratio of pZap70 to total Zap70 in computer simulations with varied Src$^{\Delta SH2\text{-}3}$ concentrations for iLID-Drop cells in dark and light states. (**b**) Ratio of pLAT to total LAT in computer simulations with varied Src$^{\Delta SH2\text{-}3}$ concentrations for iLID-Drop cells in dark and light states. (**c**) Ratio of Src concentration present within the clusters versus the surrounding cytosol as the cellular concentration of Src was raised. Light-induced positive feedback could be observed even under conditions where the fold-enrichment of clustered Src was low (< 5). (**d**) Simulation of the model under conditions where LAT was clustered without additional Zap70-LAT dimerization, revealing at least a partial signaling response (compare to **Figure 6B**).

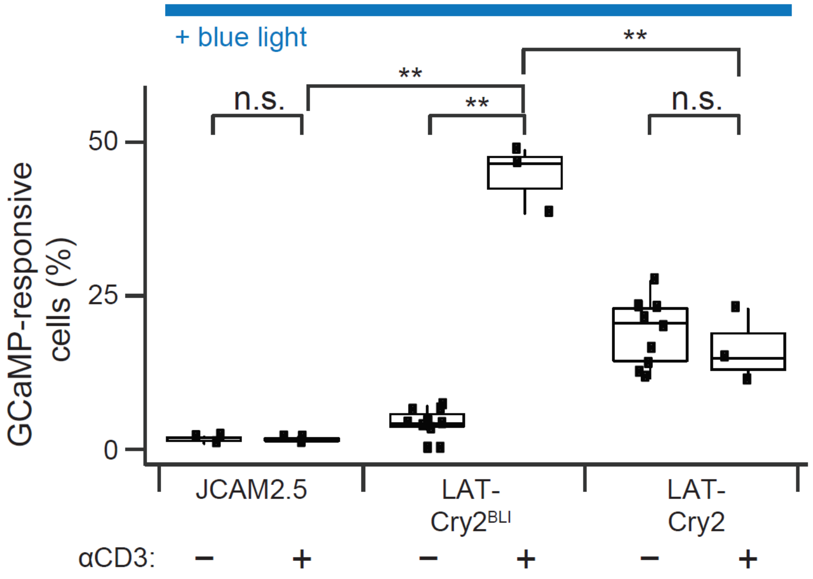

Figure S6: LAT clustering triggers light-induced signaling in LAT-deficient JCAM2.5 T cells, related to Figure 7. The fraction of GCaMP-responsive cells is plotted for three cell lines: parental JCAM2.5s and JCAM2.5s expressing either LAT-FusionRed-Cry2^{BLI} or LAT-FusionRed-Cry2. In each case, imaging was performed every 5 sec for 3 min to characterize intracellular calcium signaling using the GCaMP biosensor, in the presence or absence of an anti-CD3 crosslinking antibody. The percentage of cells exhibiting a high-amplitude, sustained increase in GCaMP intensity is shown. Each data point represents at least 50 cells quantified from a single field of view. Boxes represent median +/- quartiles. Parental JCAM2.5 cells were unresponsive to any stimulus; LAT-Cry2^{BLI} cells responded to antibody but not light, and LAT-Cry2 cells were activated by either light or antibody stimulation. n.s. = not significant; ** = $p < 0.01$.

Output	Model	Experiment
$pLAT_{lit}/pLAT_{dark}$ iLID-Only	1.68	1.47
$pZap70_{lit}/pZap70_{dark}$ iLID-Only	1.19	1.02
$pLAT_{lit}/pLAT_{dar}$ iLID-Drop	10.52	6.82
$pZap70_{lit}/pZap70_{dark}$ iLID-Drop	3.81	3.29
$pLAT_{lit}/pLAT_{dark}$ iLID-Only Zap70-K362E	2.02	3.66
$pZap70_{lit}/pZap70_{dark}$ iLID-Drop LAT FFF	1.02	1.3
$pZap70_{lit}/pZap70_{dark}$ iLID-Drop Zap70 KD	1.02	1.3
$pZap70_{lit}/pZap70_{dark}$ iLID-Drop Src ΔSH2 ΔSH3	1.02	1.38

Table S1: Comparison of model outputs to experimentally measured values. Experimental pLAT values are for Y191, related to Figure 6.

Parameter	Description	Value
$[LAT]_0$	Membrane-localized compartment concentration of LAT	0.1 µM
$[Zap70]_0$	cytosolic concentration of Zap70	1.5 µM
$[Src]_0$	cytosolic concentration of Src	0.15 µM<br>0.05 µM (ΔSH2 ΔSH3)
**K**	LAT concentration fold change upon clustering	1 (dark)<br>10 (lit, iLID-Drop)<br>1 (lit, iLID-Only)
$K_D^{iLID/SspB}$	iLID/SspB dissociation constant	13 µM (dark)<br>0.13 µM (lit, iLID-Drop)<br>0.13 µM (lit, iLID-Only)<br>from Guntas, G. et. al 2015
$K_D^{pY/SH2}$	pLAT/Src dissociation constant	0.01 µM (from Gan, 2008, and Payne et al. 1998)<br>Inf. µM (ΔSH2 ΔSH3)<br>Inf. µM (LAT FFF)
k_{cat}^{Src-a}	Catalytic rate constant for phosphorylation of Zap70 by active Src	1 s^{-1}
K_M^{Src-a}	Michaelis constant for phosphorylation of Zap70 by active Src	100 µM
k_{cat}^{Src-i}	Catalytic rate constant for phosphorylation of Zap70 by autoinhibited Src	0.05 s^{-1}<br>0.25 s^{-1} (ΔSH2 ΔSH3)
K_M^{Src-i}	Michaelis constant for phosphorylation of Zap70 by autoinhibited Src	100 µM
k_{cat}^{pZap70}	Catalytic rate constant for phosphorylation of LAT by pZap70	1 s^{-1}<br>0.25 s^{-1} (Zap70 K362E)<br>0 s^{-1} (LAT FFF)<br>0 s^{-1} (Zap70 KD)
K_M^{pZap70}	Michaelis constant for phosphorylation of LAT by pZap70	10 µM<br>200 µM (Zap70 K362E)
k^{pZap70}	Rate constant for preferential phosphorylation of LAT by tethered pZap70	0.05 s^{-1}<br>0.0125 s^{-1} (Zap70 K362E)<br>0 s^{-1} (LAT FFF)<br>0 s^{-1} (Zap70 KD)

k_n^{LAT}	Rate constant for constitutive dephosphorylation of LAT	0.01 s^{-1}
k_n^{Zap70}	Rate constant for constitutive dephosphorylation of Zap70	0.025 s^{-1}
V_{cell}	Volume of the cell	8000 μm^3
V_{mem}	Volume of the membrane-localized compartment	2400 μm^3
K_I^{PP2}	Src/PP2 dissociation constant (equal to IC50 when modeled as a non-competitive inhibitor)	0.1 μM

Table S2: Description and values of model parameters, related to Figure 6.
